# Development and mechanistic studies of calcium–BINOL phosphate-catalyzed hydrocyanation of hydrazones

**DOI:** 10.3762/bjoc.21.59

**Published:** 2025-04-14

**Authors:** Carola Tortora, Christian Andreas Fischer, Sascha Kohlbauer, Alexandru Zamfir, Gerd M Ballmann, Jürgen Pahl, Sjoerd Harder, Svetlana B Tsogoeva

**Affiliations:** 1 Department of Chemistry and Pharmacy, Organic Chemistry Chair I and Interdisciplinary Center for Molecular Materials (ICMM), Friedrich-Alexander-Universität Erlangen-Nürnberg, Nikolaus-Fiebiger Strasse 10, 91058 Erlangen, Germany; 2 Department of Chemistry and Pharmacy, Chair of Inorganic and Organometallic Chemistry, Friedrich-Alexander-Universität Erlangen-Nürnberg, Egerlandstrasse 1, 91058 Erlangen, Germany

**Keywords:** asymmetric synthesis, calcium–BINOL phosphate catalysis, hydrocyanation, hydrazones, isocyanides

## Abstract

Asymmetric hydrocyanation of hydrazones, catalyzed by a calcium–BINOL phosphate complex, has been studied for the first time both experimentally and computationally with DFT methods. A full catalytic cycle for the enantioselective synthesis of α-hydrazinonitriles is proposed based on insights gained from DFT calculations. Trimethylsilyl cyanide (TMSCN) has been used as a sacrificial cyanide source. We found that isocyanide (rather than cyanide) is a preferred coordination to calcium during the catalytic cycle, while the active catalyst prefers a side-on coordination of cyanide. The configuration-determining step is a hydrocyanation via a calcium isocyanide complex, whereas the rate-limiting step is that which recovers the calcium catalyst and replaces the TMS-bound product from the catalyst. While our experimental data demonstrate enantioselectivity values as high as 89% under certain conditions, the overall enantioselectivity achieved with the calcium catalyst remains modest, mainly due to competing pathways for the *Z*- and *E*-hydrazone isomers leading to opposite enantiomers. The experimental results confirm these computational proposals.

## Introduction

Catalytic applications of non-toxic earth abundant metals like calcium are currently on the rise [1–4]. Prominent recent examples that witness the versatility of calcium catalysis include calcium-catalyzed amination of π-activated alcohols [5], the Beckmann rearrangement under mild conditions [6], and the Nazarov-type electrocyclization of alkenyl aryl carbinols [7]. Exploiting the ease with which calcium forms hydrides, hydrogenation of aldimines, transfer hydrogenation of alkenes, and even deuteration of benzene by an S_N_Ar mechanism, have been recently achieved through calcium catalysis [8–10]. Asymmetric synthesis has also been achieved via, e.g., 1,4-addition and [3 + 2] cycloaddition of 3-tetrasubstituted oxindoles with a calcium Pybox catalyst [11–12], or through enantioselective Friedel–Crafts and carbonyl–ene reactions [13]. Since the pioneering studies by the groups of Akiyama and Terada in 2004 [14–15], many excellent results have been achieved by applying BINOL-derived phosphoric acids, which can act as proton donor and acceptor [16–19], possessing both Brønsted acid and Lewis base character [20]. Substantial effort has been invested in elucidating the mechanism by which these bifunctional compounds act as powerful catalysts [21–29]. Since Ishihara disclosed the crucial role of calcium in many purportedly purely organocatalytic BINOL phosphate-catalyzed reactions [30–31], several asymmetric synthesis applications of calcium complexes with axially chiral BINOL phosphate ligands have been reported in recent years [28,32–38], as well as complexes with other chiral phosphoric acid ligands [39]. Since then, other main group metal complexes with BINOL phosphate ligands have been discovered [40–44]. However, this catalytic system has not yet been employed explicitly in the hydrocyanation of hydrazones. In 2010, our group reported the first organocatalytic enantioselective hydrocyanation of hydrazones catalyzed by BINOL phosphate [45], giving valuable and potentially bioactive α-hydrazino acids [46–48]. This reaction has also been achieved by a lanthanide–PYBOX complex [49] and through asymmetric transfer hydrocyanation of aldimines with a boron compound [50]. Hence, we reasoned that the enantioselective hydrocyanation of hydrazones might be possible with a calcium–BINOL phosphate complex as catalyst.

Herein, we report the development of the first Ca–BINOL phosphate-catalyzed asymmetric hydrocyanation of hydrazones and the results from DFT calculations to elucidate the mechanism of this transformation.

## Results and Discussion

**Figure 1:**
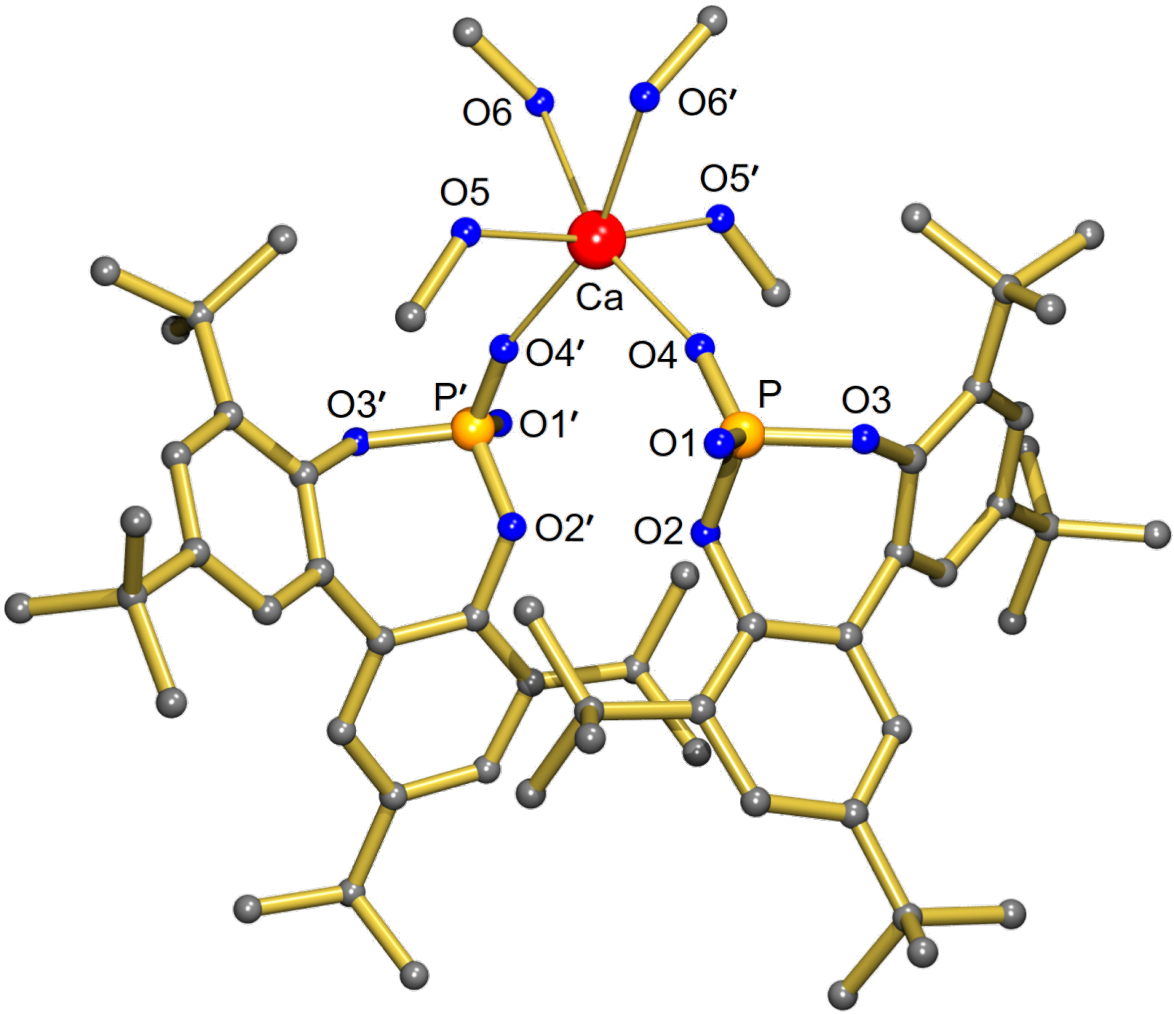
Crystal structure of the calcium diphenyl phosphate complex **4**. Hydrogen atoms are omitted for clarity. Selected bond lengths (in Å) and angle: Ca1–O4 2.2510(13), Ca1–O6 2.4004(14), Ca1–O5 2.3438(13), P–O4 1.4793(13), P–O1 1.4914(13), O4–Ca-O4′ 99.09(7)°.

**Scheme 1:**
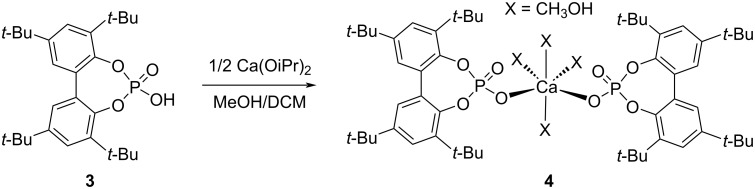
Synthesis of the calcium diphenyl phosphate model complex **4** from phosphoric acid **3** and Ca(OiPr)_2_.

We first set out to study the hydrocyanation of hydrazone **1** towards product **2** (Table 1) using an achiral model calcium-based catalyst (**4**, Figure 1) with monodentate biphenyl phosphate ligands. This model catalyst **4**, derived from the literature-known phosphoric acid BIPO_4_-H **3** [51], was synthesized by reacting **3** with Ca(OiPr)_2_ under inert conditions. The product was isolated as colorless crystals in good yield of 81% (Scheme 1). Complex **4** crystallizes as a *C*_2_-symmetric chiral mononuclear complex in which Ca is bound to two monodentate phosphate ligands (Figure 1). Four MeOH ligands complete the slightly distorted octahedral coordination sphere. The phosphate ligands are in *cis*-position with respect to each other. The structure of **4** shows similarities to that of a BINOL-derived calcium phosphate complex that also crystallizes with four methanol ligands, creating an octahedral coordination sphere with phosphate ligands in *cis*-position [52]. As observed earlier [53], *cis*/*trans* preferences in octahedral calcium complexes are influenced by small changes in sterics. Further details on the structure of **4** can be found in Supporting Information File 1.

**Table 1:** Hydrocyanation of **1** catalyzed with calcium phosphate model complex **4**.

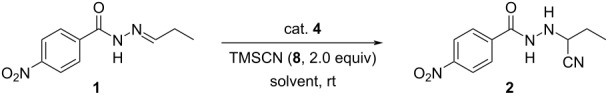				

Entry	Cat. loading [mol %]	Solvent^a^	Time [h]	Conversion [%]

1	10	DCM	2	>99
2	5	DCM	6	>99
3	2.5	DCM	12	>99
4	10	THF	12	>99

^a^DCM = dichloromethane, THF = tetrahydrofuran.

Complex **4** appeared to be a well-defined achiral model system for the catalyst combination BINOL phosphate/Ca(OiPr)_2_. In the presence of two equivalents of TMSCN, complex **4** gave a quantitative conversion to the product **2**, whereby the phosphoric acid BIPO_4_-H ligand **3** did not catalyze this hydrocyanation. At room temperature, a nearly full conversion can already be achieved within 2–12 hours, depending on the catalyst loading. This result underscores the importance of calcium in complex **4** for this particular transformation. We assume that the conversion of complex **4** to the active catalytic species might rely on the in situ formation of the cyanide species from the reaction between Ca complex **4** and TMSCN (**8**).

**Figure 2:**
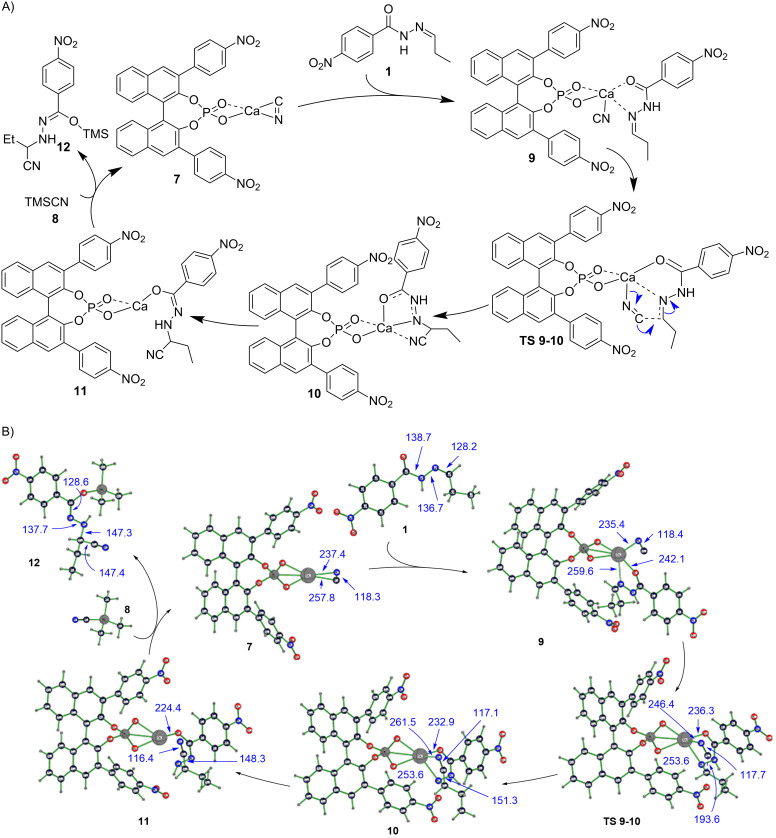
(A) Proposed catalytic cycle for the hydrocyanation of hydrazones with the Ca–BINOL phosphate catalyst. (B) Catalytic cycle for asymmetric hydrocyanation of a *Z*-hydrazone, giving the "*S*" product, catalyzed by a Ca–BINOL phosphate complex as computed at B3LYP/6-31G* level of theory. Axial chirality configuration of the BINOL phosphate is as used in experiment (i.e., "*R*"). Bond lengths and distances are given in pm. For discussion see text. Species numbers represent all respective stereoisomeric forms.

Model complex **4** initially reacts with TMSCN to give MeOTMS and HCN. Even under methanol-free conditions, an excess of TMSCN is therefore needed to achieve complete conversion (Table 1), which hints at a mechanism of catalyst activation through reaction of the metal complex with the cyanide source. This observation complies with the results from the computational study (Figure 2). In addition, some TMSCN is consumed together with an amount of BINOL phosphate through the probably parasitic formation of catalytically inactive trimethylsilyl phosphate **3a**, which we were able to characterize (see Supporting Information File 1). Unfortunately, the computationally proposed catalytically active calcium cyanide complex **7** itself could not be isolated.

Substrate conversion is retarded when the non-coordinating solvent DCM is replaced by THF (Table 1, entry 4 vs 1), which might be due to competition between Ca-hydrazone and Ca–THF binding. Considering smoothness of catalytic hydrazone hydrocyanation, similar hydrofluorination or hydroiodination seemed feasible. However, replacement of TMSCN by TMSF or TMSI did not give any product.

After establishing the activity of the model achiral catalyst **4** for non-enantioselective hydrocyanation of hydrazone **1**, we sought to demonstrate the enantioselective hydrocyanation by employing enantiopure BINOL phosphate **5** as ligand for calcium (Table 2).

**Table 2:** Asymmetric hydrocyanation of hydrazones catalyzed by calcium–BINOL phosphate complex.

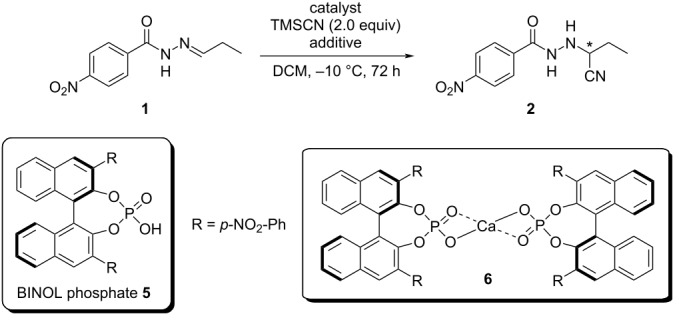					

Entry	Ca complex [mol %]	BINOL phosphate **5** [mol %]	Additive [equiv]	Yield [%]	ee [%]^a^

**1** ^b,c^	Ca(OiPr)_2_ [2.50]	5.0	*t-*BuOH [0.20]	95^d^	89 (*R*)
**2** ^b,c^	Ca(OiPr)_2_ [1.25]	5.0	*t*-BuOH [0.20]	89^d^	87 (*R*)
**3** ^b,c^	Ca(OiPr)_2_ [0.75]	5.0	*t*-BuOH [0.20]	71^d^	87 (*R*)
**4**	Cat **6** [5.0]^e,f^	–	*t*-BuOH [0.20]	88^d^	4 (*S*)
**5**	Cat **6** [5.0]^e,g^	–	phenol [2.0]	82^h^	6 (*S*)
**6**	Cat **6** [5.0]^e,g^	–	(*R*)-1-phenylethanol [2.0]	50^h^	*rac*
**7**	Cat **6** [5.0]^e,g^	–	–	92^d^	19 (*S*)
**8**	Cat **6** [5.0]^e,i^	–	–	>99^d^	19 (*S*)
**9**	Cat **6** [5.0]^e,f^	–	–	>99^d^	9 (*S*)

^a^Determined by chiral HPLC. ^b^BINOL phosphate **5** was prepared according to the procedure reported in literature [15]. After purification by column chromatography, the catalyst was washed with 2 N HCl then water, crystallized and subsequently used for the reaction. ^c^Ca complex **6** was generated in situ. ^d^Yield of isolated product. ^e^Ca–BINOL complex **6** was preformed according to the procedure employed to prepare the complex **4**. ^f^Second batch of BINOL phosphate **5** purchased by abcr GmbH and washed with 2 N HCl then water before use. ^g^BINOL phosphate **5** was purchased by abcr GmbH and directly used. ^h^Determined after calibration curve measured using compound **2** on chiral HPLC. ^i^First batch of BINOL phosphate **5** purchased by abcr GmbH and washed with 2 N HCl then water before use.

In order to compare the activity of this complex with that of BINOL phosphate **5** itself, as reported in our previous work [45], we carried out the enantioselective hydrocyanation of hydrazones using Ca–BINOL phosphate complex **6** at −10 °C in DCM for 72 h. In addition to those reaction conditions, we initially used *t-*BuOH as an additive [45]. The Ca–BINOL phosphate complex **6** was prepared in situ by reaction of the chiral ligand **5** with Ca(OiPr)_2_, varying the ratio from 2:1 to 6.6:1, respectively (Table 2, entries 1–3).

The amount of calcium salt added influences the reaction yield, which decreases when the fraction of Ca(OiPr)_2_ is lowered (Table 2, entry 3), while the good enantioselectivity remains unaffected (87–89% ee (*R-*enantiomer), entries 1–3). Because we assumed complex **6** (with two bidentate phosphate ligands) to be a precursor to the actual catalytically active species, we pre-formed it, following the procedure employed to prepare achiral complex **4** (with two monodentate phosphates, Scheme 1) and used it after isolation and characterization. In these cases, BINOL phosphate **5** was purchased, and used either without any further purification (Table 2, entries 5–7), or it was washed first with 2 N HCl solution, then rinsed with water, to remove possible traces of Ca^2+^, which could stem from industrial production (Table 2, entries 4, 8, and 9). All these experiments gave only low ee values (4–19% ee, Table 2, entries 4–9), with a preference for the *S*-enantiomer. Use of aromatic alcohol or (*R*)-1-phenylethanol as additives did not improve either yields or enantioselectivities (Table 2, entries 5 and 6 vs entry 4). Accordingly, experiments without additive performed better in terms of yield and enantioselectivity than those with additives but the ee values remain low (Table 2, entries 7–9 vs entries 4–6).

Surprisingly, in all experiments with the pre-formed catalyst **6** (Table 2, entries 4–9), a hydrocyanation product with opposite chirality was obtained, in comparison to experiments with the in situ formed Ca complex (Table 2, entries 1–3). It appears that the way in which catalyst **6** is generated (pre-formed or in situ), has a major influence on enantioselectivity, while the addition of *t*-BuOH has little to no effect (Table 2, entry 4 vs entries 1–3). This made us wonder whether the same active catalytic species is involved, when **6** is either pre-formed or presumably generated in situ.

As the mechanism of action of the calcium-BINOL phosphate catalyst remains still computationally unexplored, we set out to elucidate it by using DFT computations (Figure 2). As we have chosen as substrate in our experiments a self-prepared mixture of *E*- and *Z*-isomers (9:1) of *N*-acyl hydrazone **1**, which allows for additional binding to oxophilic calcium via the carbonyl oxygen, we did not know a priori which of the two isomers undergoes the hydrocyanation reaction more facile and therefore we studied both reaction pathways for the *Z*- and for the *E*-hydrazone. The assumed resting state of the catalyst is complex **7** (Figure 2), in which BINOL phosphate serves as a bidentate ligand to calcium in a pseudo-tetrahedral coordination environment with cyanide, stemming from a common reagent (vide infra) and binding to the metal in a preferred side-on (π-complex) mode [54].

**Figure 3:**
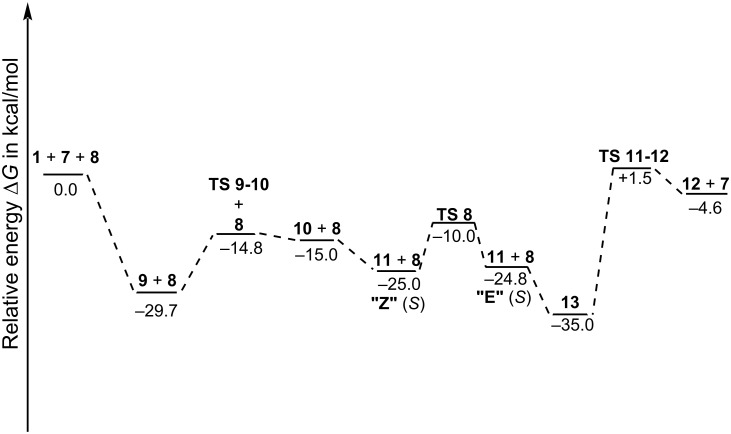
Reaction energy profile for the hydrocyanation of *Z*-hydrazone **1**, (depicted is the pathway that gives the "*S*" product), catalyzed by in situ-formed calcium BINOL phosphate complex **7**, as computed at the B3LYP/6-31G* level of theory, including zero-point energy, thermodynamic, and dispersion energy corrections (with Grimme's semiempirical D3 correction and Becke–Johnson damping, cutting off terms with higher than r^−6^ asymptotic dependence).

When the hydrazone substrate enters the catalytic cycle, it coordinates also as a bidentate ligand to calcium via oxygen and nitrogen atoms, resulting in detachment of the cyanide carbon from the calcium atom to yield an isocyanide complex **9** [55], in which the preferred side of attack on the imine carbon of the hydrazone is already predetermined (i.e., from either *Re* or *Si* face). Notably, in both complexes formed from *E*- and *Z*-hydrazone, an attack from the *Si* side is favored. Formation of hydrazone complex **9** is strongly exothermic (Figure 3), rendering this reaction step effectively irreversible. Relative stabilities of diastereomers for *Z*- and *E*-hydrazones play therefore a minor role, as relative amounts of the four possible stereoisomers of **9** (for *Z*- and *E*-configuration, and for *Re* or *Si* side attack) should be more or less equal and the actual stereoselectivity must hence be determined in later stages of the reaction.

Ligand sites in the pre-complex **9** form a pseudo-tetragonal pyramid, with isocyanide at the apex. Atoms in the Ca–NC moiety are non-linear with a Ca–N–C angle of 100°. Due to the stereospecificity of the reaction, racemization would have to occur already in **9** through a topomerization (e.g., similar to a Berry pseudorotation) of the metal complex, considering that species with a quaternary carbon, as they appear in later stages of the catalytic cycle, are less likely to racemize without bond breaking. The barrier to pyramidal inversion has been found to be 15.1 kcal·mol^−1^. Berry rotation itself proceeds via a tetragonal pyramid as transition-state structure and does therefore not occur here. A second isocyanide isomer, with a more acute Ca–N–C angle of 92°, has greater resemblance to a (here absent) side-on form (found to dominate computationally for Ca(CN)_2_ in the gas phase) [54]. Fortunately, that isomer in which the isonitrile carbon is closer to the imine carbon (i.e., the hydrocyanation reaction center), is 0.5 kcal·mol^−1^ lower in energy. Substrate **1** may conceivably undergo a tautomerization towards an iminol form, 14.2 kcal·mol^−1^ higher in energy. Hence, amounts of this isomer are negligible in equilibrium and do not play a role also in the formation of the pre-complex **3**.

The competing cyanide form of **9** (with a collinear arrangement of Ca–C–N) was found to be a sizable 3.3 kcal·mol^−1^ higher in energy than the preferred isonitrile, conforming to earlier anticipations based on computations of alkaline earth metal dicyanides [54]. Similarly, it also has been recently observed that boron-catalyzed transfer hydrocyanation of alkenes proceeds via (boron) isocyanides [50].

If calcium cyanide would be preferred over isocyanide, hydrocyanation would hardly be possible. A principally conceivable "hydroisocyanation" of hydrazone is therefore precluded. The relative stability of alkaline earth metal cyanides versus isocyanides had been subject of high-level quantum chemical computations [54], and has gained interest lately [56–58].

Thanks to preferred isocyanide (rather than cyanide) coordination to calcium, the carbon atom in **9** has increased nucleophilicity, compared to that in **7** – an important provisioning for the following reaction step. Hydrocyanation hence proceeds facially with a barrier of only 10.2 kcal·mol^−1^ via the configuration-determining **TS 9-10** by attack of isocyanide at the imine carbon of hydrazone. If the configuration-determining step would be also rate limiting, experimental observation of enantioselectivity must be explained by relative energies of stereoisomeric forms of **TS 9-10**. Reversible formation of product **10** from nucleophilic addition step via **TS 9-10** is slightly endothermic (Figure 3). In **10**, cyanide is still attached via nitrogen to calcium, forming a formal Ca–N–C–C–N–(Ca) five-ring. This initial product is consumed in a tautomerization step to give slightly more stable **11**, in which the cyanide group is no longer bound to the calcium atom. Tautomerization **10** → **11** (with N_β_ hydrogen shifting to N_α_, the former imine nitrogen) is most likely intermolecular, as direct [1,2]H shift is orbital symmetry forbidden and has, hence, a high barrier of 39.8 kcal·mol^−1^, and also because a – conceivable – intermittently at carbonyl oxygen protonated species (i.e., on an iminol-type pathway) is considerably higher in energy than **10** or **11**.

The TMS-bound product **12**, proposed in the computational investigation, could not be isolated either – probably due to its high sensitivity to hydrolysis. However, a 2D ^1^H-^15^N NMR correlation spectrum showed no interactions of hydrogen nuclei in silicon-bound methyl groups and any of the nitrogens in the product. This led us to conclude that silicon is bound via the carbonyl oxygen in **12**, as also shown by DFT computations. This is further corroborated by observation of a ^13^C NMR signal at 146 ppm for an sp^2^ carbon in the backbone of the product, which fits better for a C=N than a C=O bond.

Recovery of the catalyst resting state **7** is achieved by reacting **11** with stoichiometric reagent TMSCN (**8**). This "reloads" calcium with cyanide and replaces TMS-bound isolated product **12** from the metal complex and in which silicon, rather than calcium, is bound to the carbonyl oxygen. The addition reaction itself is rather exothermic (Figure 3) and provides (together with the similarly exothermic complexation of calcium complex **7** with hydrazone) driving force for the catalytic cycle, but, on the other hand, may impede enantioselectivity, because of a "backwater" effect equaling out outcomes from different reaction pathways before intermediates are allowed to overcome the rate-limiting barrier. The actual replacement step itself (Figure 5), which recovers the catalyst and releases product **12**, is endothermic and slow (Figure 3). The overall reaction energy of the catalytic cycle is, hence, a mere 4.6 kcal·mol^−1^, taking into account zero-point energy and thermodynamic corrections. Getting acceptable yields experimentally at all is only possible because of swiftness and practical irreversibility of formation of new **9** from released **7**. Furthermore, it could be helpful to constantly remove the product from the reaction zone.

**Figure 4:**
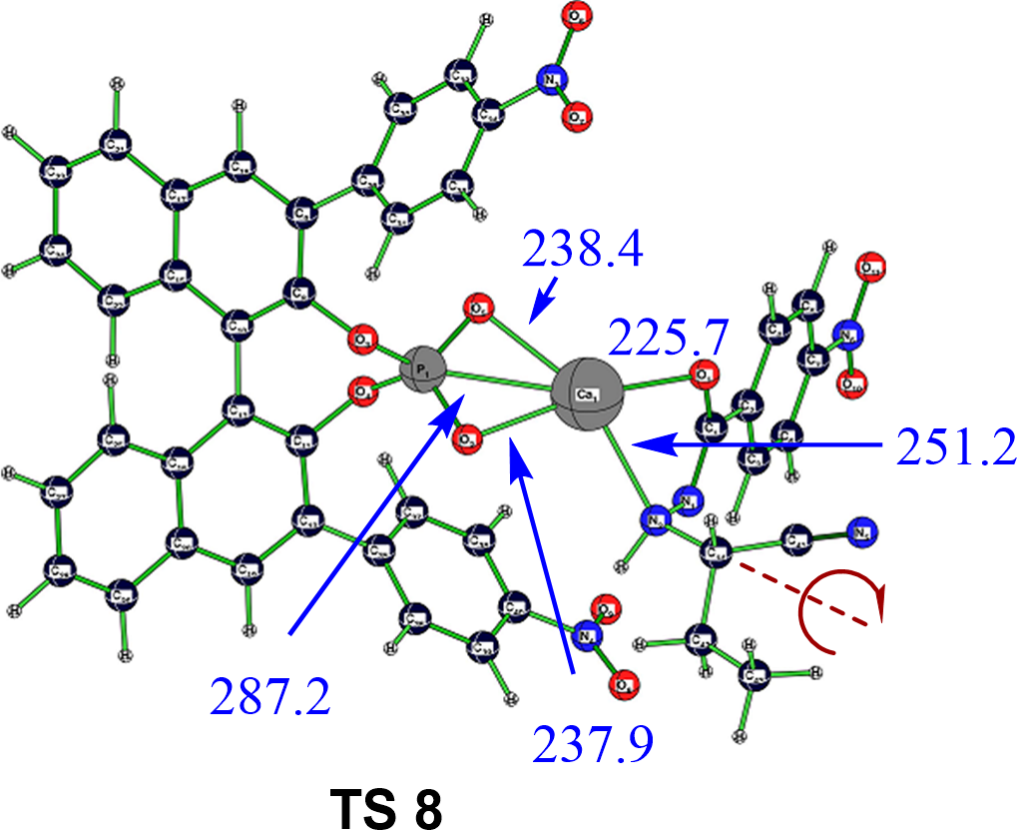
Transition-state structure **TS 8** for internal rotation, mixing conformational (*Z*/*E*)-pathways with opposite enantioselectivity. Bond lengths and distances are given in pm.

Moreover, the replacement step will occur only from a conformation in which the ethyl group at the former imine carbon stands opposite to the TMS moiety. While the CN double bond of imine **1** became a single bond in complexes **10** and **11** through the hydrocyanation step, internal rotation around this bond is still massively hindered with a sizable barrier of 15.0 kcal·mol^−1^ via **TS 8** (Figure 4), interchanging two almost equienergetic conformations of **11** (**11Z**, with an N–N–C–C_Et_ torsion angle of 32°, and **11E**, with an torsion angle of 111°), which have a strong reminiscence of their predecessors' former geometric isomerism, an effect that might similarly occur also in related addition reactions of alkenes, for example.

After **11E** had reacted with TMSCN, adduct **13** forms, 3.8 kcal·mol^−1^ more stable than the competing unreactive adduct obtained from "*Z*" conformation.

Outside from the catalytic cycle, **12** could be further hydrolyzed in a subsequent step to yield hydrazino nitrile **2** (Table 2) from which α-hydrazino acids could be obtained by harsher hydrolysis conditions. It is important to note that compound **12** also exhibits geometric isomerism (Figure 2 depicts the lower-energy configuration), which arises from the newly formed CN double bond.

One of the most important quantities which allows comparison with experimental data is enantioselectivity. The most favorable **TS 9-10** is that which leads to the "*S*"-configured product, when the substrate is *Z*-hydrazone. Product of opposite handedness is reached via a transition state, 1.0 kcal·mol^−1^ higher in energy, resulting in a theoretical ee value of 74% (at 263 K), if pure *Z*-hydrazone would be used and under the assumption of kinetic reaction control. In contrast, the *E*-hydrazone gives preferably the "*R*" product (with a theoretical ee value of 64%). The transition state which gives the *R*-product from *E*-hydrazone is slightly lower in energy (Table 3).

**Table 3:** Relative energies (in kcal·mol^−1^) at B3LYP/6-31G* for different pathways involving the configuration-determining hydrocyanation step. Absolute configuration (*R* or *S*) refers to that in hydrocyanation product **10**.

Pathway	*E*_rel_ of **9**	*E*_rel_ of **TS 9-10**	*E*_rel_ of **10**

*Z* (*S*)	1.6	11.8	6.0
*Z* (*R*)	2.6	12.8	6.9
*E* (*S*)	0.0	11.7	5.1
*E* (*R*)	0.3	10.9	4.2

However, the activation barrier for the rate-limiting step is 0.4 kcal·mol^−1^ lower for the pathway shown in Figure 2 and that yields the *S*-product from the *Z*-hydrazone. As the activation barrier to thermal *E*/*Z*-isomerization is more than twice as high as the barrier to hydrocyanation [59], the initial *E*/*Z* ratio is mostly conserved and affects strongly the experimentally achievable ee value, which must necessarily be much lower than the theoretical values because of the opposite handedness of products preferred for *E*- and *Z*-hydrazones, respectively.

**Figure 5:**
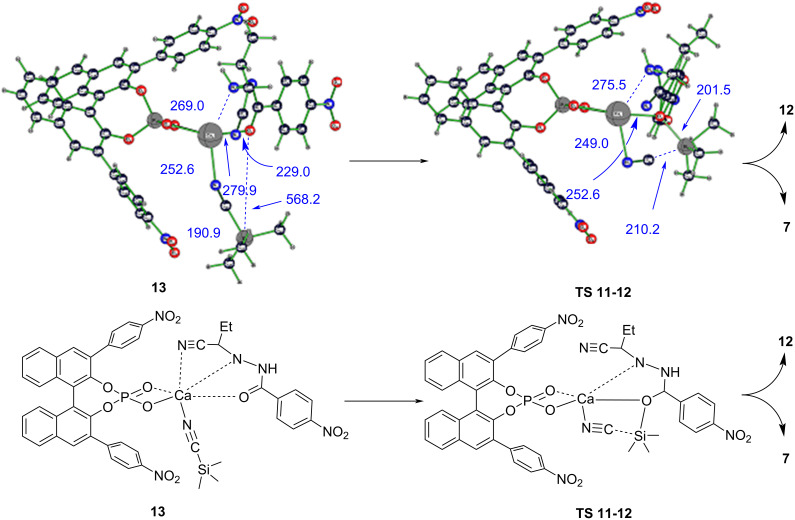
Replacement step after internal rotation in **11** via **TS8** and reaction with TMSCN to give adduct **13** (see Figure 3). Distances and bond lengths are given in pm. Catalyst **7** is replaced in **TS 11-12** by concerted electrophilic intramolecular substitution with formal trimethylsilyl cation as electrophile. Please note the surprisingly short Ca···N distance to the product nitrile nitrogen atom in **13**.

A thermodynamic control of the reaction outcome (i.e., especially enantioselectivity) would require the possibility to approach an equilibrium between **9** and **10** (seeing the reversibility of step **9** → **10**), in which case the relative energies of stereoisomeric forms of **10** determine the experimentally achievable ee values. Maximal theoretical ee values are 70% "*S*" for *Z*-hydrazone and 70% "*R*" for *E*-hydrazone. The relatively high racemization barriers in **9** preclude a Curtin–Hammett scenario (with fast pre-equilibrium), which would otherwise render the enantiomeric outcome (ee value) independent of the relative stabilities of different forms of this complex. Consequently, under kinetic reaction control, enantioselectivity would decrease over time as the reaction progresses. This occurs because slower-reacting complexes would accumulate, leading to a gradual equilibration of reaction rates for the *S*- and *R*-forming stereoisomers of **9**. The final step in the catalytic cycle (Figure 5), i.e., replacement step **13** → **7**, is rate limiting in calcium–BINOL phosphate-catalyzed asymmetric hydrocyanation of hydrazones and proceeds via a transition-state structure **TS 11-12**, 1.5 kcal·mol^−1^ higher in energy than that of the entry channel (**1** + **7** + **8**, Figure 3). Because the rate-determining and configuration-determining steps are different, reaction control of the whole multistep transformation in terms of enantioselectivity is therefore neither predominantly thermodynamic nor predominantly kinetic. However, trends in (and even extent of) enantioselectivity are very similar for both kinetic and thermodynamic control for the configuration-determining hydrocyanation step and for both *Z*- and *E*-hydrazones, respectively.

The replacement step is initiated by forming an adduct **13** from reaction of **11E** with TMSCN (**8**), giving a rather low energy complex as an energetic "sink" in the mechanism and with an almost linear Si–CN–Ca chain with a bridging cyanide ligand (Figure 5).

Catalyst **7** is released in the subsequent endothermic step via **TS 11-12**, which also yields the TMS-bound product **12**. In this transition-state structure, two bonds (Ca–O and Si–C) are broken, while a Si–O bond is simultaneously formed, in a concerted step implying a cyclic flow of six electrons in a five-membered ring. The isocyanide Ca–N–C bond angle in **TS 11-12** lies between that of **7** and **9**. While the replacement step is slow, and hence, the bottleneck of the hydrocyanation, it is practically irreversible because the released free catalyst **7** is rapidly engaged again by reaction with further hydrazone in a distinctly exothermic reaction step.

## Conclusion

In summary, we demonstrated that calcium-BINOL phosphates are able to catalyze the hydrocyanation reaction of hydrazones, towards precursors of α-hydrazino acids. Model achiral catalyst **4**, pre-formed by reaction of Ca(OiPr)_2_ and biphenyl phosphate **3**, led to racemic product **2** in nearly full conversion in different reaction times, depending on the amount of catalyst used. Biphenyl phosphate **3** alone did not catalyze the reaction, showing that the catalytically active species is formed in situ by reaction between the Ca complex **4** and TMSCN. We also performed the reaction using chiral Ca complex **6**, either formed in situ or pre-formed as mentioned above for **4**, using enantiopure BINOL phosphate **5** as ligand. By generating the pre-catalyst **6** directly in the reaction vessel, we proved the involvement of Ca^2+^ in the catalysis, since the yield decreases by reducing the amount of Ca(OiPr)_2_ with respect to the BINOL phosphate **5**. When Ca complex **6** is prepared and isolated prior to the reaction, use of alcohols as additives has a negative effect on both yield and enantioselectivity. The enantioselectivity was generally low (Table 2, entries 4–9) and with preference for the *S*-configured hydrocyanide employing the pre-formed Ca complex **6**. In addition, product **2** was obtained with absolute configuration opposite to the results with in situ-formed Ca complex **6**, for which BINOL phosphate was newly synthesized, instead of purchased, and with which we achieved much higher ee values (87–89% ee (*R*), Table 2, entries 1–3). These distinctly different outcomes, when methods to generate Ca complex **6**, or source and purification of ligand **5**, are varied, showed that these parameters strongly influence the hydrocyanation enantioselectivity.

In order to elucidate the mechanism of action of the pre-catalyst complex **6** and to explain the observed low enantioselectivity, we have carried out DFT computations with both *E*- and *Z*-hydrazone **1**, which revealed that Ca–BINOL phosphate complex **7** with side-on-coordinated cyanide is likely to be the active catalytic species and catalyst resting state. This complex reacts with hydrazone to give an encounter complex **9** with end-on-bound isocyanide, that attacks the imine carbon in the hydrazone to give species **10** in which cyanide is bound to both calcium and the imine carbon and which readily rearranges to cyanide **11**. This process is only slightly faster for the *Z*-hydrazone than for the *E*-hydrazone. Moreover, according to computations, the *Z*-hydrazone gives preferentially the *S*-, whereas the more stable *E*-hydrazone gives preferentially the *R*-configured product. However, in order to replace TMS-bound product **12** from the calcium complex and retrieve catalyst **7**, prior internal rotation in **11** from *Z*-hydrazone pathway to *E*-hydrazone pathway is required, i.e., both hydrazone isomers give the same hydrocyanation product and with low ee, because pathways with opposite preference for either product enantiomer mix via internal rotation **TS8**, which explains also the low enantioselectivity observed in our experiments and with preformed pre-catalyst **6**. Which absolute configuration prevails eventually in the product (i.e., *R* or *S*), is difficult to predict theoretically due to the fact that the configuration and rate-determining steps are not the same.

The good enantioselectivities observed when the catalytic species was presumed to be in situ-generated pre-catalyst **6**, indicate that catalysis must have followed therein a route different from the one with preformed pre-catalyst **6**, and involves a different active species. Seeing that calcium is prone to form complexes with mono- and bidentate phosphate ligands, bridged multicentered particles may form in solution during in situ generation of the calcium complex, as known from, e.g., Posner's well-defined colloidal calcium phosphates [60].

## Supporting Information

CCDC 1824279 (**4**), CCDC 1824280 (**3**), and CCDC 1824281 (product O=C(C1=CC=C(Br)C=C1)NNC(C#N)CC2=CC=CC=C2, **2’’**) contain the supplementary crystallographic data for this paper. These data are provided free of charge by The Cambridge Crystallographic Data Centre via http://www.ccdc.ac.uk/data request/cif.

File 1 Synthetic procedures, ^1^H, ^13^C, and ^31^P NMR as well as mass-spectrometric data of all synthesized compounds and selected crystal structures.

## Data Availability

Data generated and analyzed during this study is available from the corresponding author upon reasonable request.
