# Supplementary material for: Development and mechanistic studies of calcium–BINOL phosphate-catalyzed hydrocyanation of hydrazones
Source: Beilstein J Org Chem. 2025 Apr 14;21:755–65. doi: 10.3762/bjoc.21.59 (PMC12018919; doi:10.3762/bjoc.21.59)
Supplement: File 1 — Synthetic procedures, 1H, 13C, and 31P NMR as well as mass-spectrometric data of all synthesized compounds and selected crystal structures. [file Beilstein_J_Org_Chem-21-755-s001.pdf]

BEILSTEIN JOURNAL OF ORGANIC CHEMISTRY

Supporting Information

for

Development and mechanistic studies of calcium–BINOL phosphate-catalyzed hydrocyanation of hydrazones

Carola Tortora, Christian A. Fischer, Sascha Kohlbauer, Alexandru Zamfir, Gerd M. Ballmann, Jürgen Pahl, Sjoerd Harder and Svetlana B. Tsogoeva

Beilstein J. Org. Chem. **2025,** *21,* 755–765. doi:10.3762/bjoc.21.59

Synthetic procedures, ^{1}H, ^{13}C, and ^{31}P NMR as well as mass-spectrometric data of all synthesized compounds and selected crystal structures

License and Terms: This is a supporting information file under the terms of the Creative Commons Attribution License (https://creativecommons.org/licenses/by/4.0). Please note that the reuse, redistribution and reproduction in particular requires that the author(s) and source are credited and that individual graphics may be subject to special legal provisions.
The license is subject to the *Beilstein Journal of Organic Chemistry* terms and conditions: (https://www.beilstein-journals.org/bjoc/terms)

Table of contents

1. General method and materials S2

2. Computational methods S3

3. Syntheses and catalytic procedures S4

4. Monitoring the reaction between complex **4** and TMSCN by ^{31}P NMR S8

5. Characterization of the raw product **12’** from catalytic conversion of hydrazone with TMSCN S10

6. NMR-spectra of **4** and **3a** S14

7. Crystal structure data S18

8. References S27

1. General method and materials

All metal organic experiments were conducted under an inert nitrogen atmosphere by applying standard Schlenk techniques or using nitrogen-filled gloveboxes (Firma MBraun, Labmaster SP). Organic reactions were carried out under air if not indicated differently. Solvents for metal organic reactions were either dried over sodium metal and subsequently distilled (THF), degassed with nitrogen, and dried over activated aluminum oxide (Innovative Technology, Pure Solv 400-4-MD, Solvent Purification System) (pentane, hexane) or dried over MS 3 Å and degassed with nitrogen (DCM, MeOH). All other solvents and commercially available chemicals were used as received without further purification.

NMR spectra were recorded with a Bruker Avance III HD 400 MHz or 600 MHz NMR spectrometer. Chemical shifts (δ) are given in ppm (parts per million) values, coupling constants (*J*) in Hz (hertz). For describing signal multiplicities common abbreviations are used: s (singlet), d (dublet), t (triplet), q (quartet), sept. / hept. (septet or heptet), m (multiplet) and br (broad). Spectra were referenced due to solvent residual signal [1]. Deuterated solvents were purchased from Sigma Aldrich or Euriso-top, stored over MS 3 Å and degassed with nitrogen prior use.

Single crystals have been measured on a SuperNova A (Dual) diffractometer with Atlas S2 CCD Nova (Cu) and Mova (Mo) X-ray sources,

GC–MS measurements were performed on a Thermo Scientific™ Trace™ 1310 gas chromatography system (carrier gas helium) with detection by a Thermo Scientific™ ISQ™ LT Single Quadrupole mass spectrometer.

Elemental analysis was performed with a Hekatech Eurovector EA3000 analyzer.

2. Computational methods

We used density functional theory for geometry optimization without any geometry restriction and computation of properties with the GAUSSIAN09 quantum chemistry program suite [2]. Unless otherwise stated, B3LYP/6-31G* level had been used throughout, with Becke's three parameter functional [3] and the correlation correction of Lee, Yang and Parr [4], unless otherwise stated. Pople's et al. valence split basis sets have been used throughout [5]. All stationary points on their respective potential energy surfaces (PES) have been rigorously characterized by the number of imaginary frequencies in harmonic frequency calculations. Relative energies are zero-point energy (ZPE) corrected. Transition-state structures have been matched to their pertaining minimum structures by an intrinsic reaction coordinate (IRC) following algorithm. In order to take into account dispersion energy effects, Grimme's D3 semi-empirical dispersion energy correction has been used, together with Becke–Johnson damping, to facilitate convergence [6]. Cartesian coordinates of all species can be requested from the authors.

3. Syntheses and catalytic procedures

Syntheses according to literature:

Hydrazone (**1**):

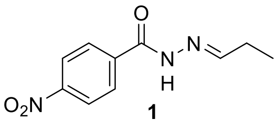

1

The compound was synthesized according to literature procedure [7].

BINOL-phosphoric acid (**5**):

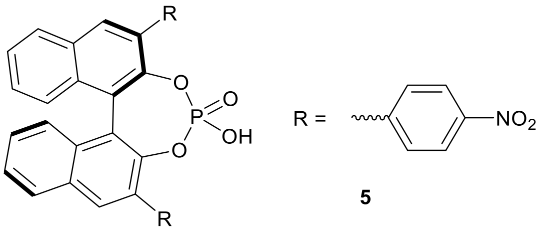

5

The compound was synthesized according to literature procedure [8].

Tetra-*tert*-butyl biphenyl phosphoric acid (**3**):

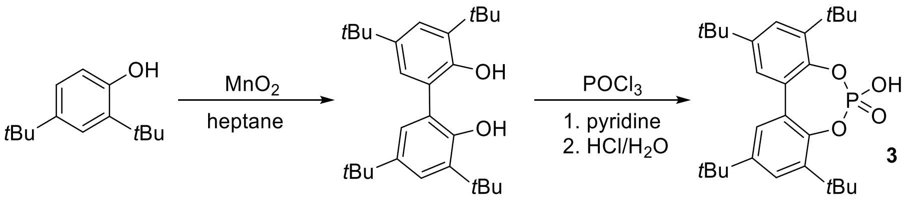

3

Biphenol: was synthesized according to literature procedure [9].

Phosphoric acid was synthesized according to literature procedure [10].

Details for the crystal structure determination of structure of **3** can be found below.

Synthetic procedures:

Calcium di-(tetra-*tert*-butylbiphenyl phosphate) complex **4**:

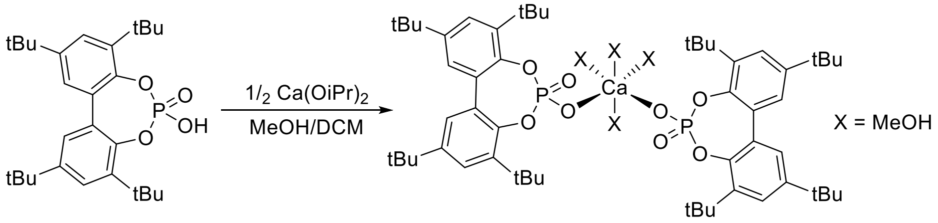

3 **4**

Phosphoric acid **3** (500 mg; 1.06 mmol) and $Ca(OiPr)_2$ (83.7 mg; 0.530 mmol) were dissolved in DCM (5 mL) and MeOH (5 mL) resulting in a clear solution. After a short time, a precipitate formed and the resulting suspension was stirred overnight. After removal of the solvent the crude product was extracted with MeOH (2 × 10 mL). Solvent removal and drying in vacuum led to a white solid which was identified as complex **4** (487 mg; 0.430 mmol; yield 81%). All four MeOH ligands can be removed by heating the complex under high vacuum (10^{-6} mbar) at 60 °C for 3 hours.

Analysis: (DMSO was used as co-solvent to increase solubility of the complex in pure DCM)

^{1}H NMR (600 MHz, DCM-d_2) δ (ppm) 7.39 (s, 4H, Ar), 7.09 (s, 4H, Ar), 1.49 (s, 36H, CCH_3), 1.30 (s, 36H, CCH_3).

^{13}C NMR (151 MHz, DCM-d_2) δ (ppm) 147.5 (Ar), 145.8 (Ar), 140.7 (Ar), 132.3 (Ar), 126.1 (Ar), 124.5 (Ar), 35.8 (*C*CH_3), 34.8 (*C*CH_3), 31.9 (CCH_3), 31.8 (CCH_3).

^{31}P NMR (243 MHz, DCM-d_2) δ (ppm) -2.3.

Anal. Calc. for $C_{56}H_{80}CaO_8P_2$ + 4 CH_3OH: C, 64.84; H, 8.71. Found: C 65.19, H 8.50.

Trimethylsilylphosphate **3a**:

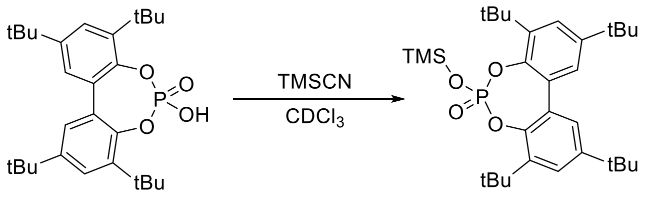

3 **3a**

Under nitrogen atmosphere phosphoric acid **3** (21 mg; 0.044 mmol) was weighed into an NMR tube and dissolved in $CDCl_3$ (600 µL). TMSCN (4.4 mg; 5.6 µL; 0.044 mmol) was added. Instantly, the product **3a** and HCN could be detected by NMR methods (conversion >99%). Removal of the solvents led to a white powder. A large-scale synthesis using TMSCl in pyridine was also conducted and a clean reaction leading to product **3a** was observed, however problems in purification (i.e. removal of pyridine) did not lead to an analytically pure substance.

Analysis:

^{1}H NMR (600 MHz, $CDCl_3$) δ (ppm) 7.49 (s, 2H, Ar), 7.20 (s, 2H, Ar), 1.51 (s, 18H, CCH_3), 1.35 (s, 18H, CCH_3), 0.19 (s, 9H, $SiCH_3$).

^{13}C NMR (151 MHz, $CDCl_3$) δ (ppm) 147.8 (Ar), 144.8 (Ar), 140.2 (Ar), 130.5 (Ar), 126.7 (Ar), 125.2 (Ar), 35.6 (CCH_3), 34.9 (CCH_3), 31.6 (CCH_3), 31.3 (CCH_3), 0.7 ($SiCH_3$).

^{31}P NMR (243 MHz, $CDCl_3$) δ (ppm) -11.9.

GC/MS (EI-MS 70 eV): m/z: 544.34 $[C_{31}H_{49}O_4PSi]^+$, 529.28 $[C_{30}H_{46}O_4PSi]^+$, 471.24 $[C_{28}H_{40}O_4P]^+$; t_R: 15.50 min.

Catalytic procedures:

Chiral catalysis benchmark

To a flask containing the catalyst (**5** or complex **4;** 0.0136 mmol), a solution of hydrazone **1** (0.136 mmol) in dichloromethane (3 mL) and *t*-BuOH (0.027 mmol; 20 mol %) was added at −10 °C. After stirring for 20 min, TMSCN (0.272 mmol; 2 equiv) was added to the reaction mixture and stirring was continued for 72 h at −10 °C. Temperature control was achieved by the use of a cryostat. The reaction mixture was directly applied on a silica gel column and purified by chromatography (EtOAc/DCM/PE 4:1:5).

Comparison of the obtained NMR signals was in agreement with earlier published data [7]. In the experiments conducted with a chiral calcium phosphate, ee values were determined by HPLC: Diacel Chiralpak IB, *n*-hexane/isopropanol 90:10, flow rate 1 mL/min, λ = 254 nm, t_{R1} = 12.8 min (*R*), t_{R2} = 24.4 min (*S*).

Ca(BINOL-phosphate **5**)$_2$: BINOL-phosphoric acid **5** (8.0 mg; 0.0136 mmol) and Ca(OiPr)$_2$ (1.1 mg; 0.0068 mmol) were dissolved under inert conditions in a mixture of 1 mL dichloromethane and 1 mL methanol for 1 h at 20–25 °C. The solvents were removed under vacuum, 2 mL dichloromethane was added and removed under vacuum two times. The obtained oil was dried under high vacuum (approx. 3 mbar) at room temperature. The catalytic reactions were performed in the same flask.

NMR-scale catalysis

Hydrazone **1** (0.0041 mmol) and the respective amount of catalyst **4** (2.5, 5, or 10 mol %) was dissolved with the specific amount of *t*-BuOH (0, 10, or 20 mol %) in DCM-d_2. Directly after the addition of TMSCN (0.082 mmol; 2 equiv) a ^{1}H NMR spectrum of the sample was recorded. Conversion was monitored by periodic ^{1}H NMR measurements until no hydrazone was detectable anymore. Finally, hydrolysis of the samples resulted in the corresponding product **2** with NMR resonances equal to those reported previously [7].

Attempts to crystallize the raw product of catalytic transformations (i.e. before hydrolysis) by slow evacuation of the solvent in a nitrogen atmosphere failed. The crystals obtained were found to be the hydrolysis product **2** (vide infra for further details).

4. Monitoring the reaction between complex 4 and TMSCN by ^{31}P NMR

The reaction between catalyst **4** and TMSCN in DCM-d_2 was followed by ^{31}P NMR spectroscopy. Immediately after sample preparation a broad ^{31}P resonance at −4.45 ppm was found (Figure S1). Cooling the NMR sample from 298 K to 243 K led to decoalescence into three major signals. The signal at −3.3 ppm is assigned to a phosphate ligand bound to Ca (cf. the phosphate ligands in **4** in DCM-d_2 show a ^{31}P resonance at −2.3 ppm). The very broad signal at −9.9 ppm is assigned to TMS-phosphate **3a** coordinated as a neutral ligand to Ca (cf. **3a** in $CDCl_3$ shows a ^{31}P resonance at −11.9 ppm). A sharper signal at −7.2 ppm is assigned to the phosphate ligand in (phosphate)CaCN·(TMS-phosphate).

We interpret these results with the following conversion:

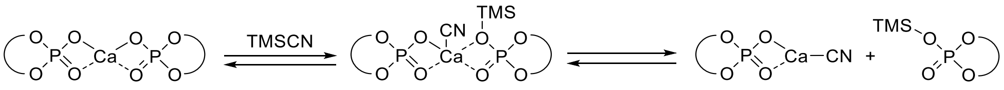

4 **3a**

Monitoring catalytic experiments by ^{31}P NMR shows a similar situation. The amount of free TMS-phosphate **3a** increases during the catalytic experiment. Finally all phosphate ligands have been converted to free **3a** (Figure S5) suggesting that all Ca is converted to insoluble $Ca(CN)_2$:

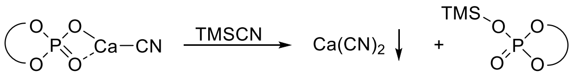

3a

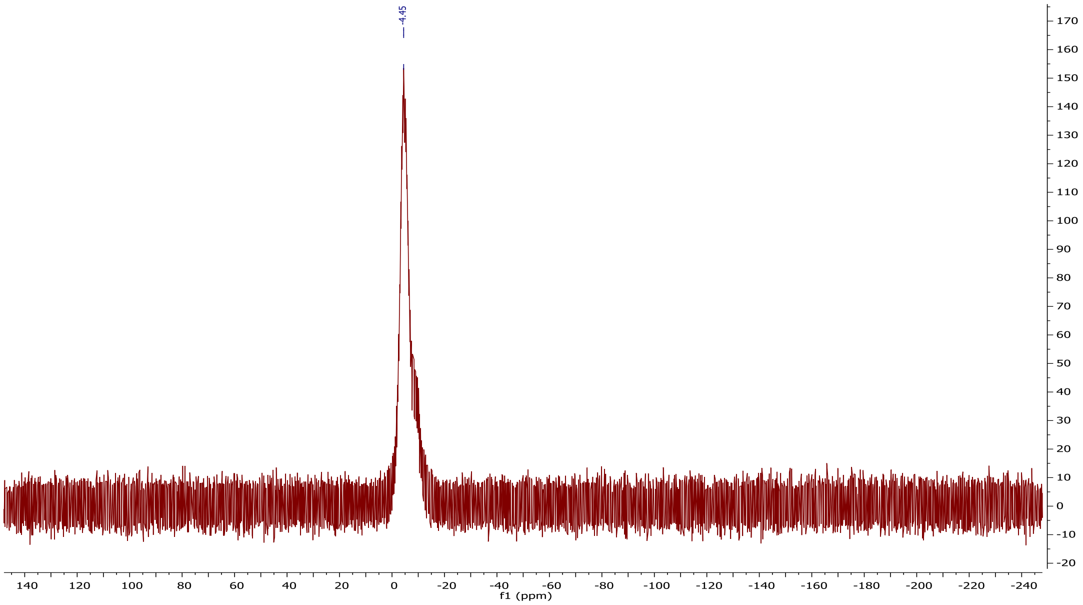

Figure S1. ^{31}P NMR-spectra of complex **4** with TMSCN at 298 K (162 MHz, DCM-d_2).

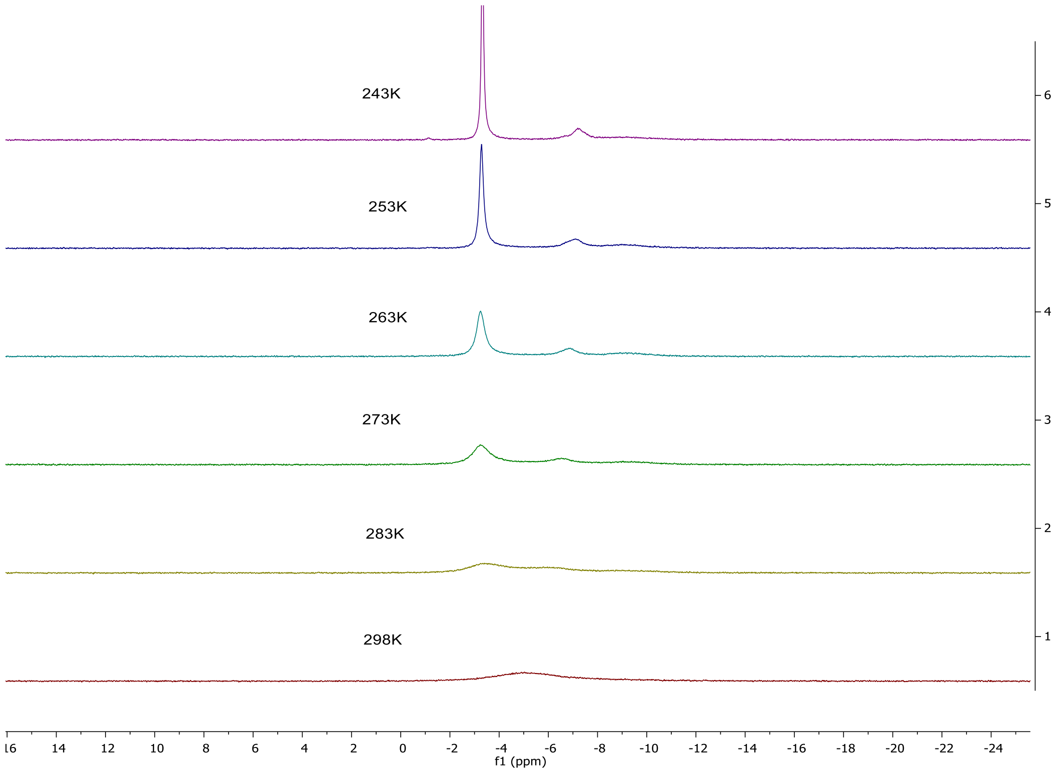

Figure S2. ^{31}P NMR-spectra of complex **4** with TMSCN at variable temperatures (298 K to 243 K) (162 MHz, DCM-d_2).

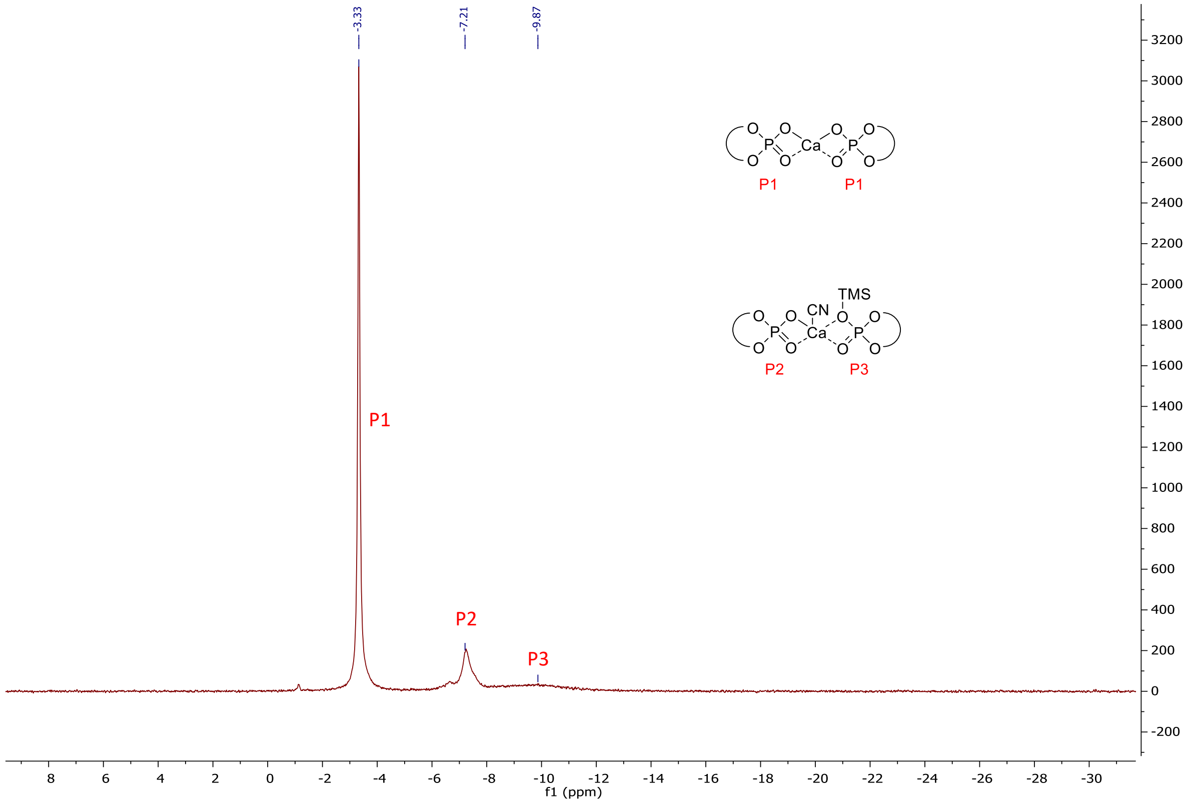

Figure S3. ^{31}P NMR spectru of complex **4** with TMSCN at 243 K (162 MHz, DCM-d_2).

5. Characterization of the raw product 12’ from catalytic conversion of hydrazone with TMSCN

The Et substituent in hydrazone **1** was replaced with a benzyl substituent (this gives fewer NMR signals in the aliphatic range facilitating NMR assignments). After catalytic transformation with TMSCN, the reaction mixture was analyzed by ^{1}H, ^{13}C, ^{31}P, ^{1}H-^{15}N HSQC and ^{1}H-^{15}N HMBC NMR (Figure S4–S8). The product was characterized as silylether **12’**. Hydrolysis of the sample resulted in the corresponding product **2’** with NMR resonances equal to those reported earlier [7].

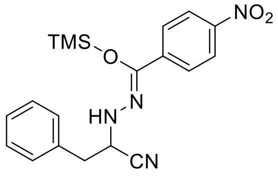

12'

NMR data for **12’**

^{1}H NMR (600 MHz, DCM-d_2) δ 7.45 – 7.36 (m, 2H, Ar), 7.35 – 7.28 (m, 2H, Ar), 7.18 – 7.15 (m, 2H, Ar), 7.15 – 7.12 (m, 3H, Ar), 5.10 (d, *J* = 7.1 Hz, 1H, N*H*), 4.20 (q, *J* = 7.1 Hz, 1H, C*H*), 3.10 – 2.98 (m, 2H, CH_2), 0.00 (s, 9H, SiCH_3).

^{13}C NMR (151 MHz, DCM-d_2) δ 146.4 (*C*O), 136.0 (*C*N), 133.5 (*Ar*), 132.0 (*Ar*), 130.1 (*Ar*), 129.4 (*Ar*), 128.5 (*Ar*), 128.1 (*Ar*), 124.1 (*Ar*), 120.3 (*Ar*), 53.4 (*C*H), 38.5 (*C*H_2), 0.9 (Si*C*H_3).

P = signals of the converted substrate; C = signals of the degraded catalyst

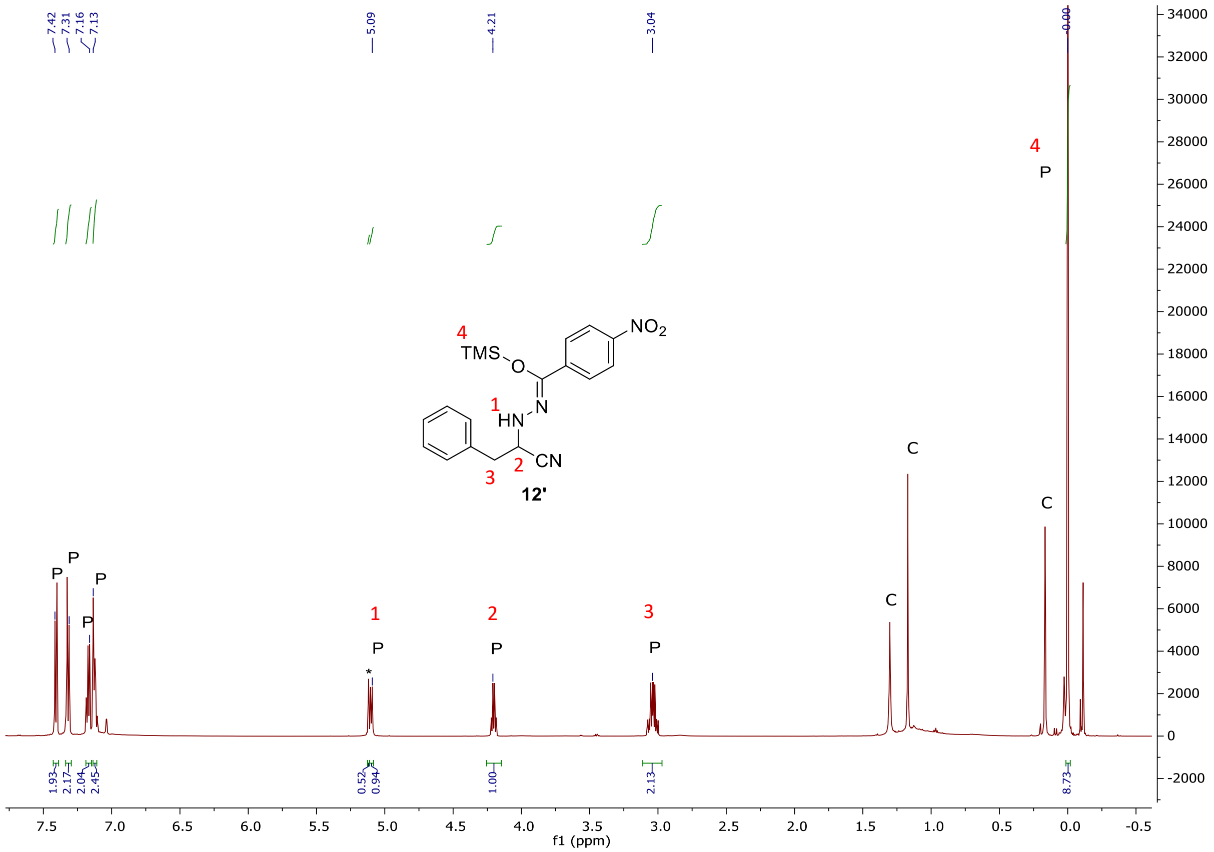

Figure S4. ^{1}H NMR spectrum at the end of catalysis (600 MHz, DCM-d_2).

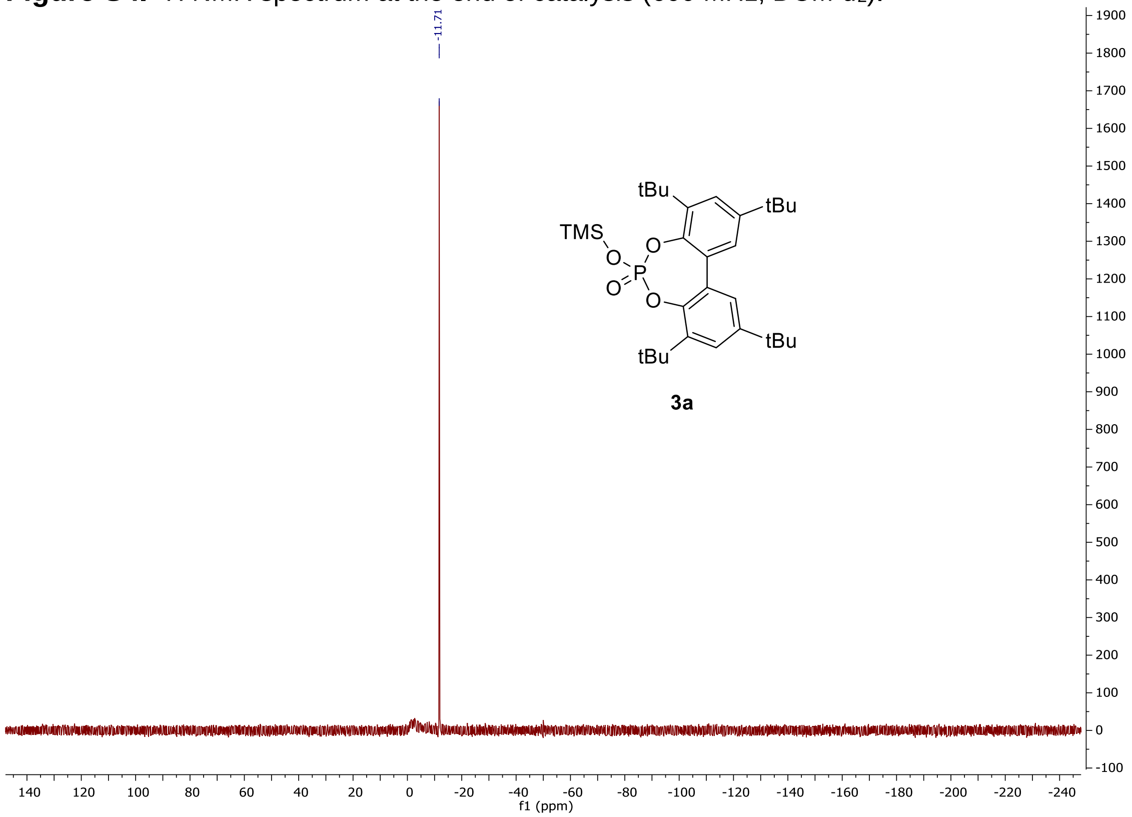

Figure S5. ^{31}P NMR spectrum at the end of catalysis (243 MHz, DCM-d_2).

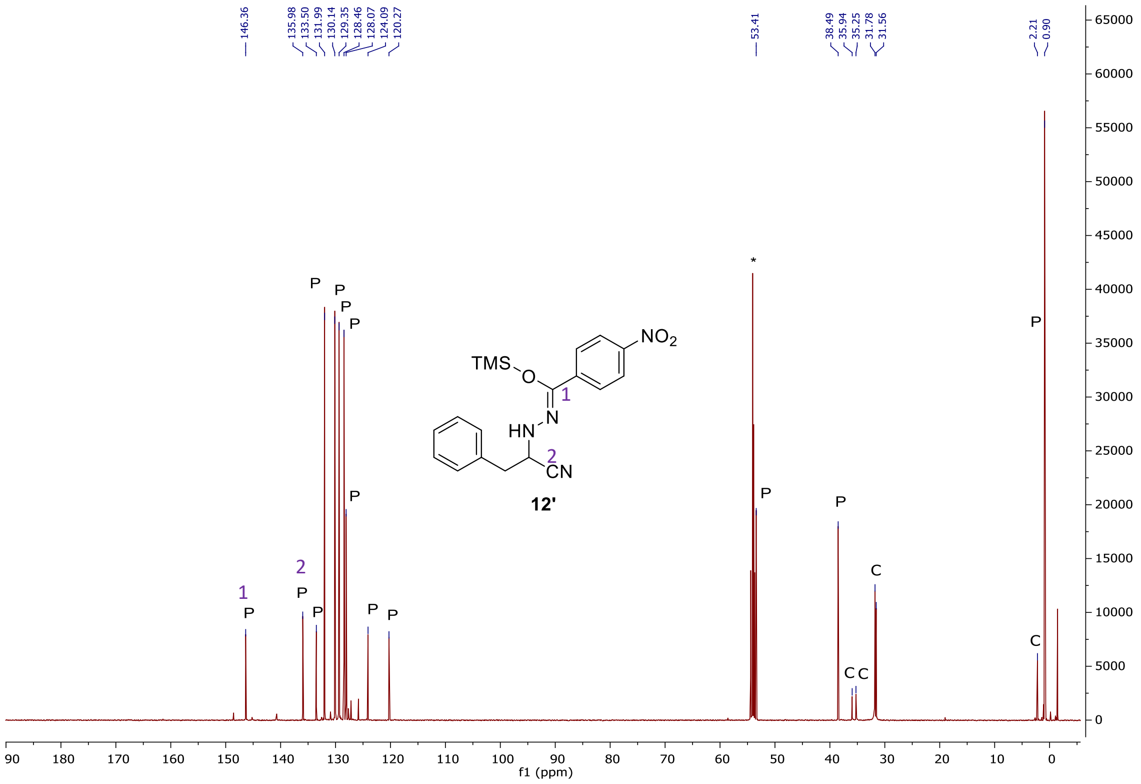

Figure S6. ^{13}C NMR spectrum at the end of catalysis (151 MHz, DCM-d_2).

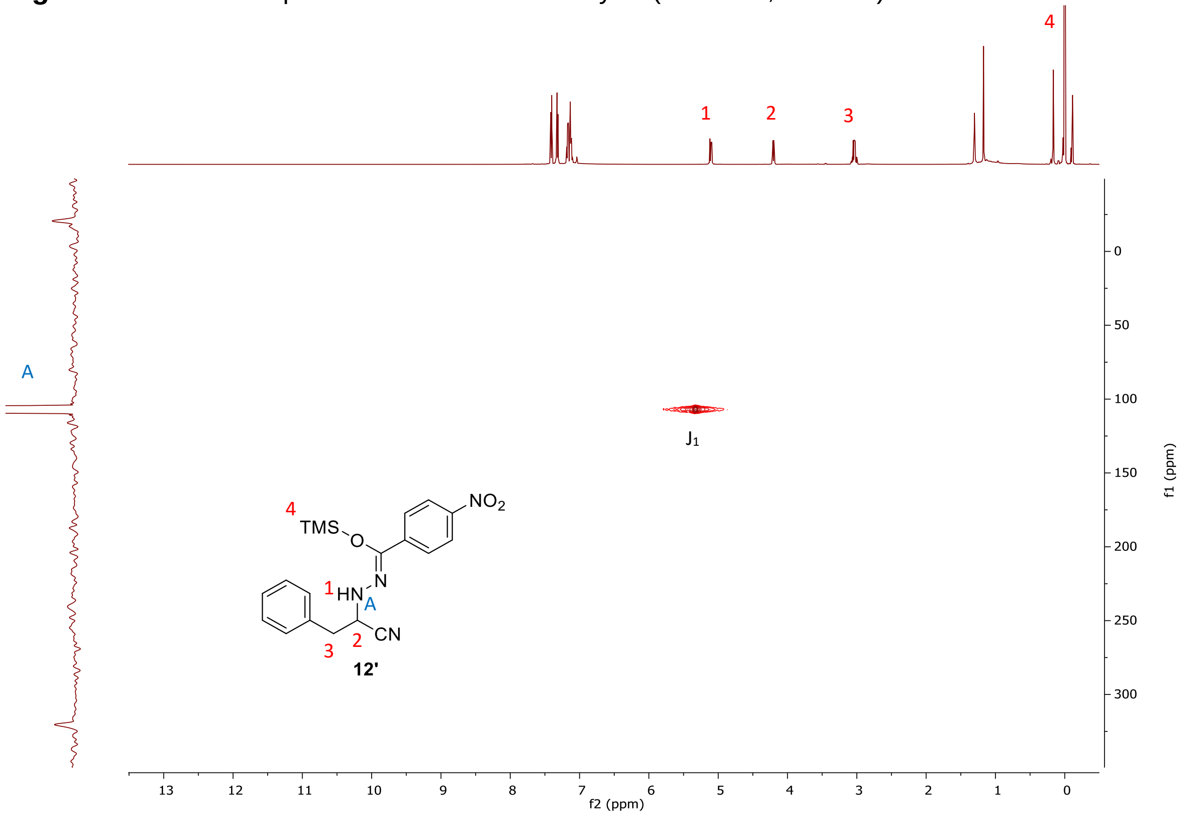

Figure S7. ^{1}H-^{15}N HSQC NMR spectrum at the end of catalysis (600 MHz, DCM-d_2).

4

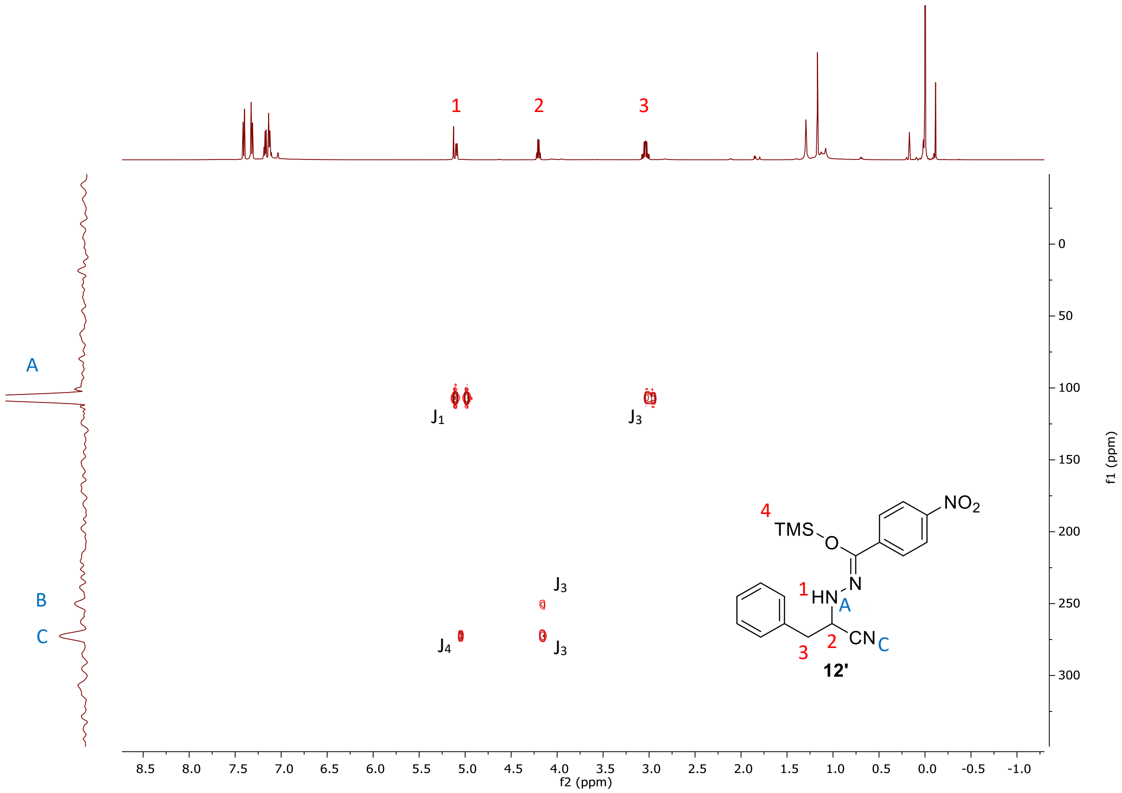

Figure S8. ^{1}H-^{15}N HMBC NMR spectrum at the end of catalysis (600 MHz, DCM-d_2)

6. NMR spectra of 4 and 3a

NMR spectra for complex Ca(phosphate)$_2$ (**4**):

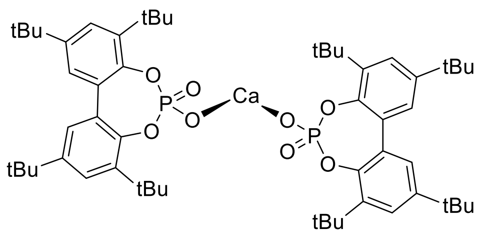

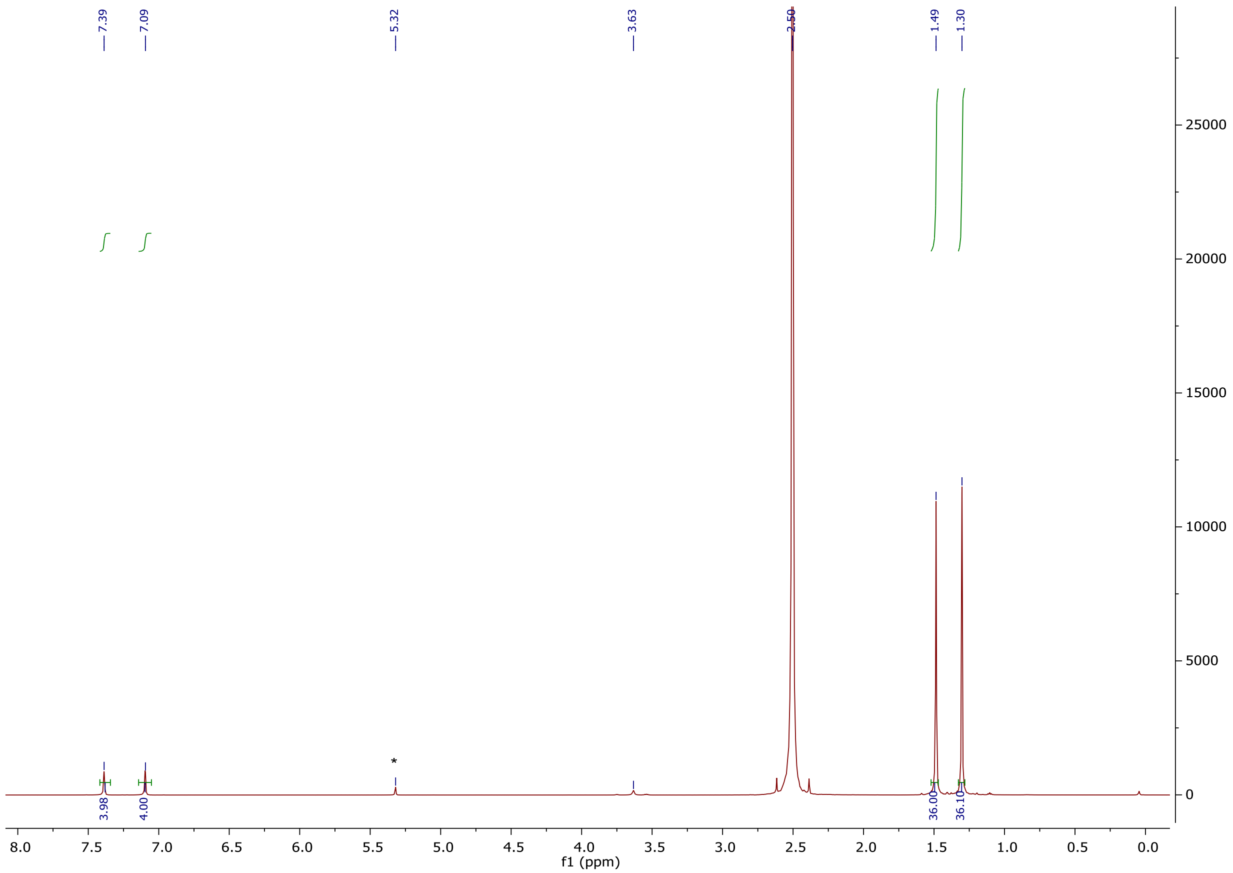

Figure S9. ^{1}H NMR spectrum of complex **4** without coordinated MeOH (600 MHz, DCM-d_2) (DMSO was used as co-solvent to increase solubility of the complex in pure DCM).

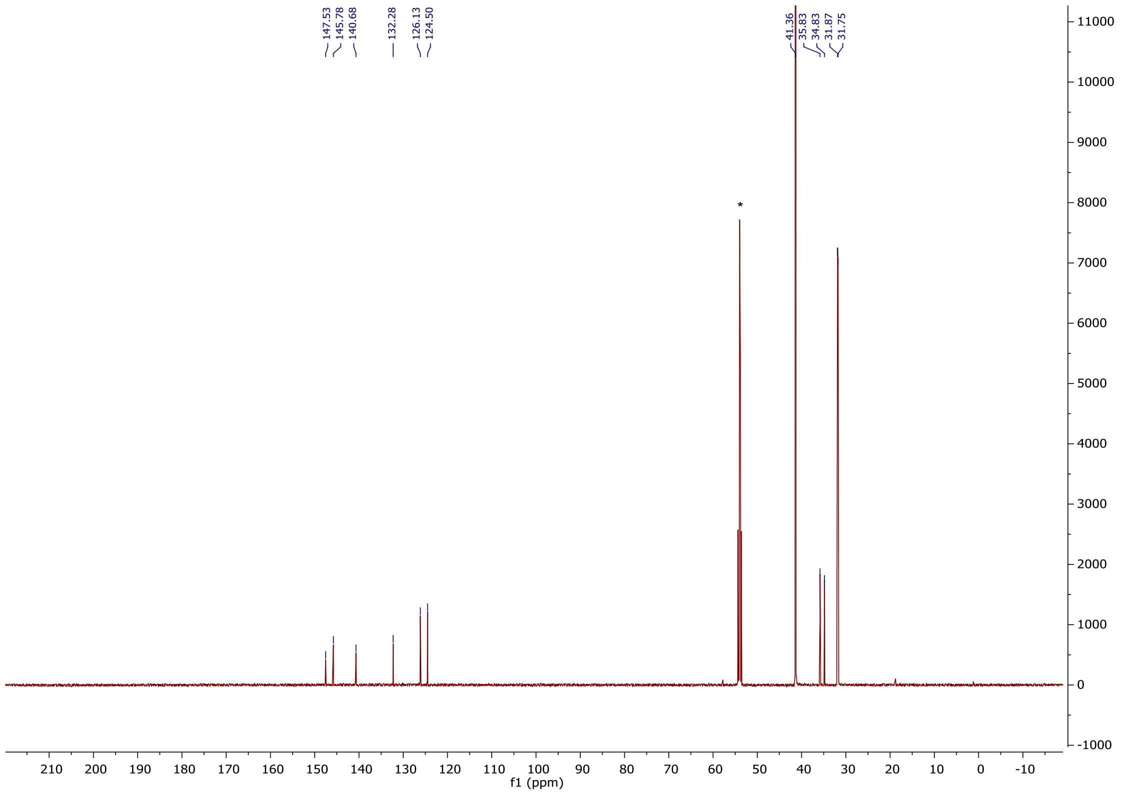

Figure S10. ^{13}C NMR spectrum of complex **4** without coordinated MeOH (151 MHz, DCM-d_2) (DMSO was used as co-solvent to increase solubility of the complex in pure DCM).

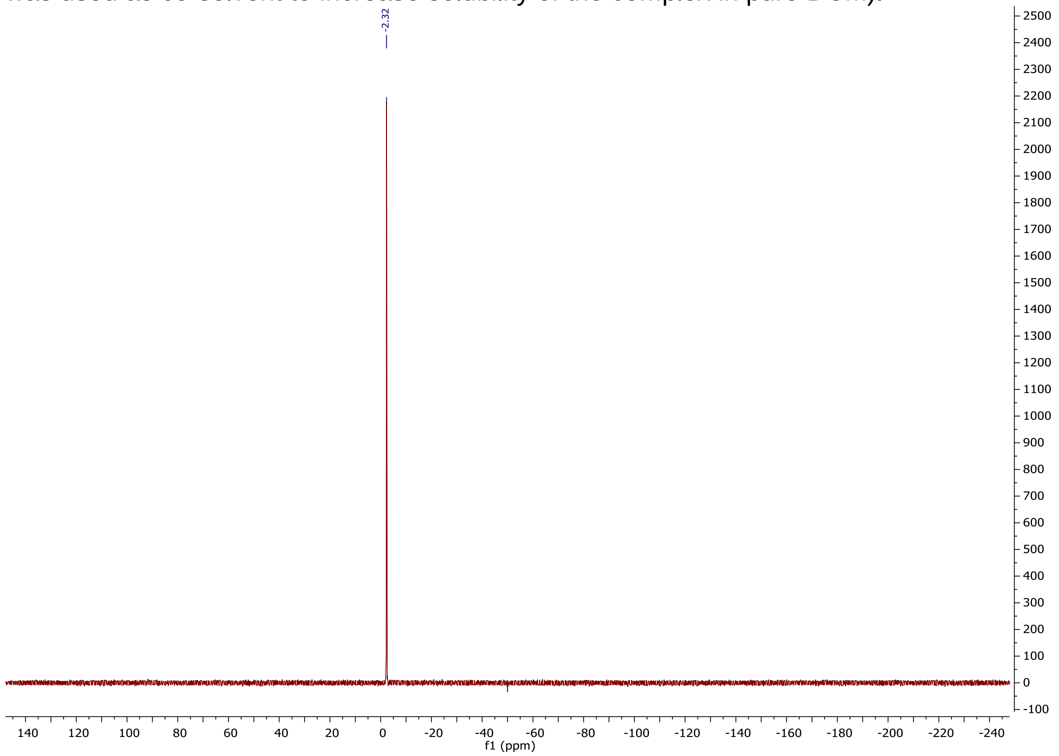

Figure S11. ^{31}P NMR spectrum of complex **4** without coordinated MeOH (243 MHz, DCM-d_2) (DMSO was used as co-solvent to increase solubility of the complex in pure DCM).

NMR spectra for trimethylsilylphosphate **3a**:

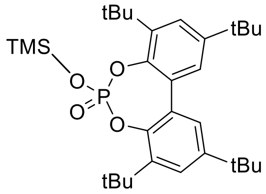

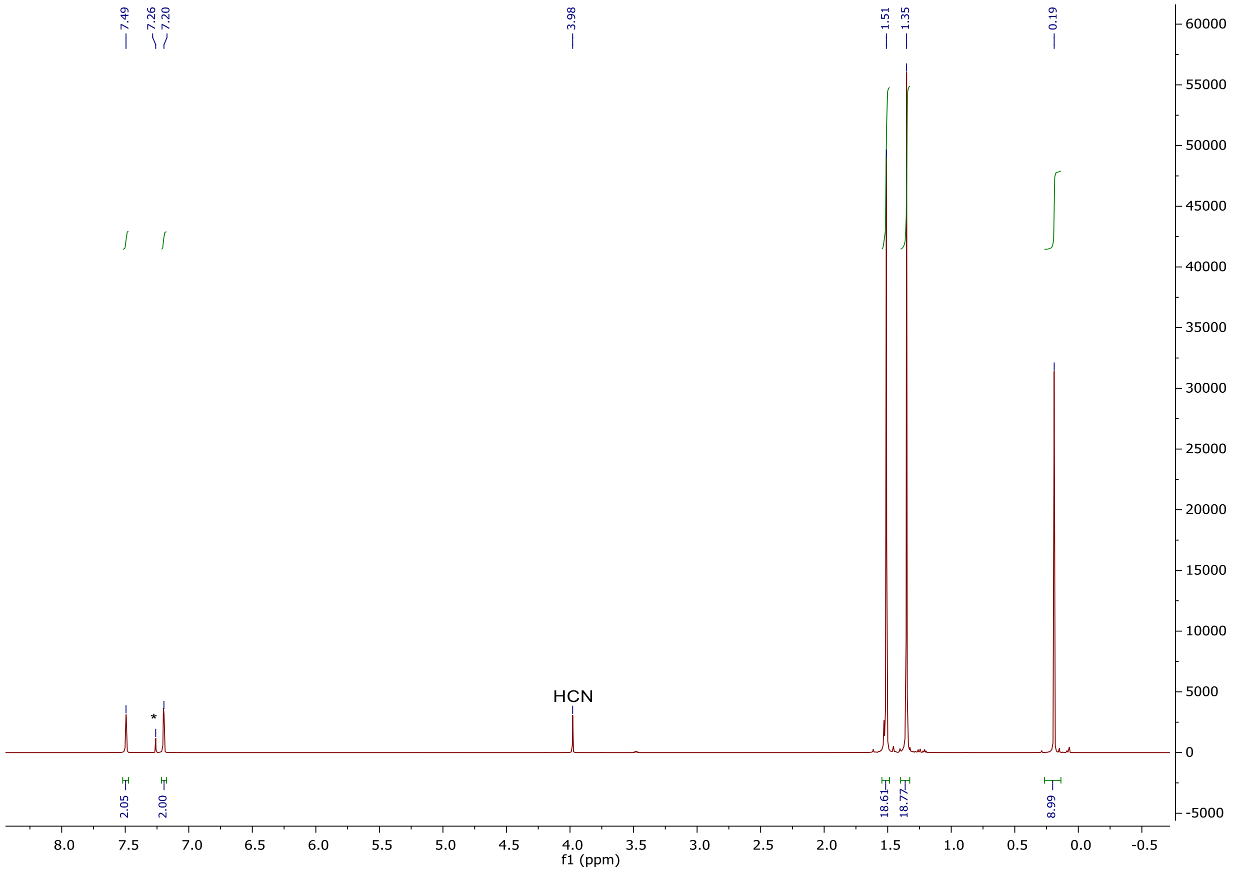

Figure S12. ^{1}H NMR spectrum of compound **3a** prepared from phosphoric acid **3** and TMSCN (600 MHz, $CDCl_3$).

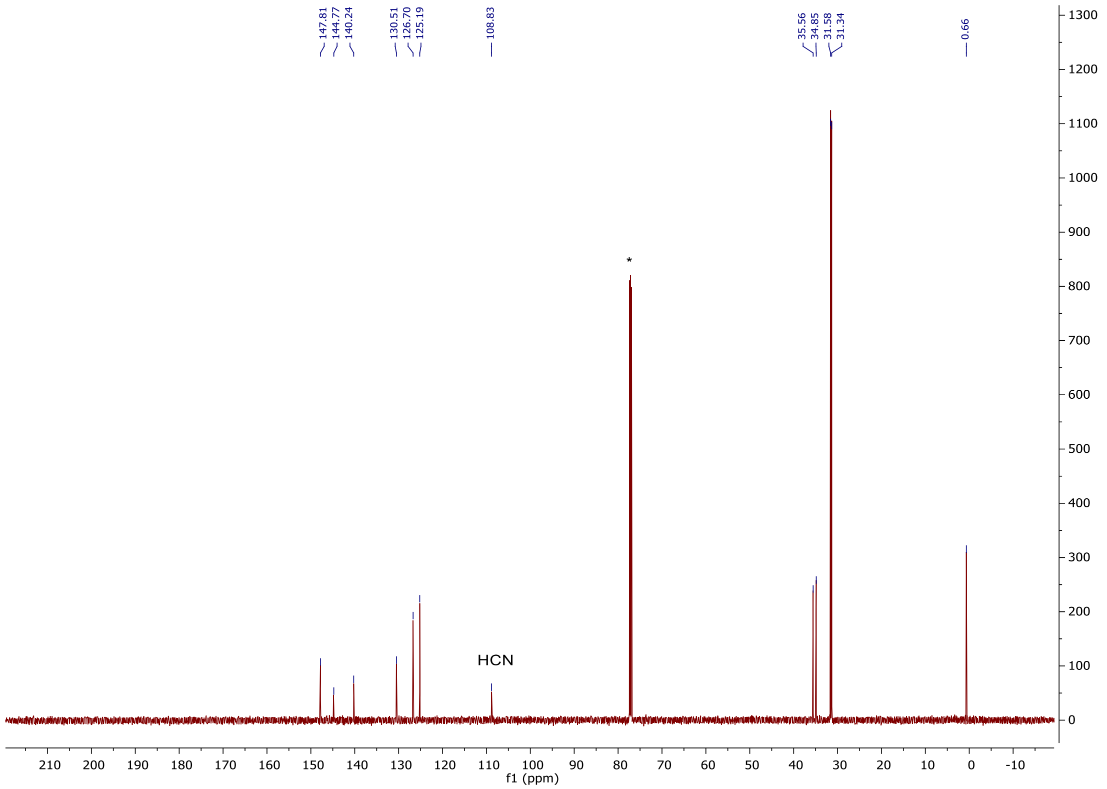

Figure S13. ^{13}C NMR spectrum of compound **3a** (151 MHz, $CDCl_3$).

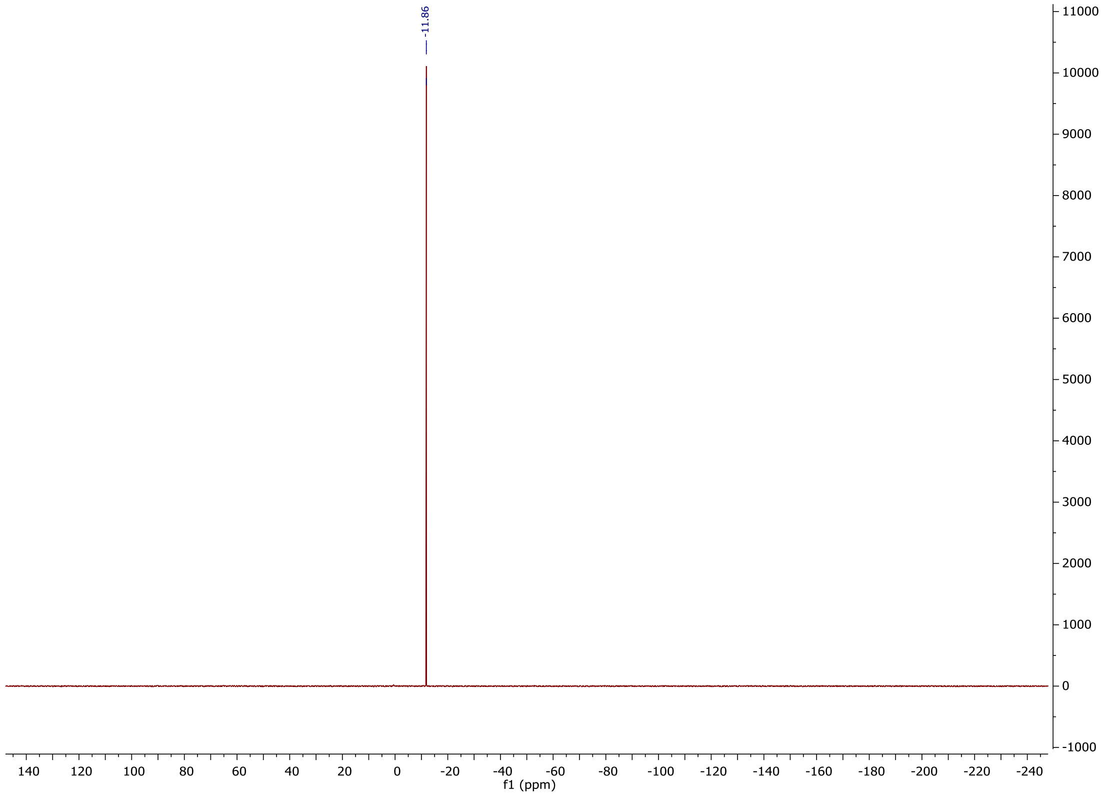

Figure S14. ^{31}P NMR spectrum of compound **3a** (243 MHz, $CDCl_3$).

7. Crystal structure data

Calcium di(tetra-*tert*-butylbiphenyl phosphate) complex **4** – CCDC 1824279

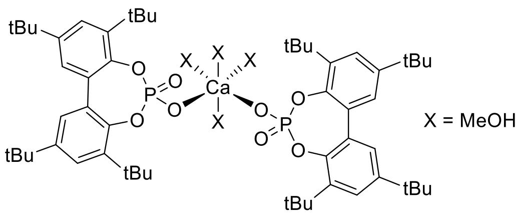

4

Using Olex2 [11], the structure was solved by direct methods (ShelXT)[12] and refined with ShelXL [13] using least squares minimization. The hydrogen atoms have been placed on calculated positions and were refined isotropically in a riding model. One *tert*-butyl substituent has two disordered positions each with an approximate ratio of 90:10. The less occupied part of the *tert*-butyl substituent was modeled with similar bond (SIMU) restraints and equalization of ADPs (EADP).

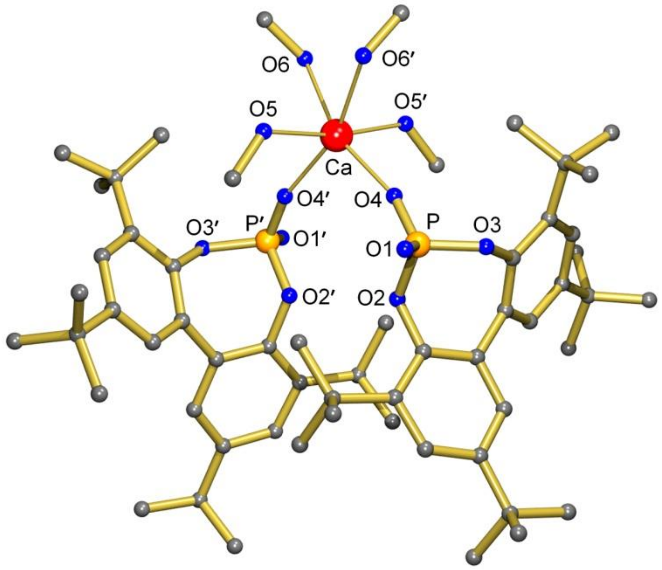

Figure S15. Crystal structure of **4**; hydrogen atoms omitted for clarity.

Table S1. Crystal data and structure refinement for complex **4**.

Identification code	hasj151111c
Empirical formula	$C_{62}H_{100}CaCl_4O_{12}P_2$
Formula weight	1281.23
Temperature/K	100
Crystal system	monoclinic
Space group	I2/a
a/Å	12.64832(9)
b/Å	18.88693(15)
c/Å	29.4527(2)
α/°	90
β/°	93.0793(7)
γ/°	90
Volume/Å^3	7025.74(9)
Z	4
ρ_{calc}g/cm^3	1.211
μ/mm^{-1}	3.035
F(000)	2744.0
Crystal size/mm^3	0.2135 × 0.122 × 0.1021
Radiation	CuKα (λ = 1.54184)
2Θ range for data collection/°	8.422 to 136.224
Index ranges	-15 ≤ h ≤ 15, -22 ≤ k ≤ 22, -35 ≤ l ≤ 35
Reflections collected	60031
Independent reflections	6401 [R_{int} = 0.0473, R_{sigma} = 0.0190]
Data/restraints/parameters	6401/12/412
Goodness-of-fit on F^2	1.061
Final R indexes [I>=2σ (I)]	R_1 = 0.0439, wR_2 = 0.1281
Final R indexes [all data]	R_1 = 0.0456, wR_2 = 0.1303
Largest diff. peak/hole / e Å^{-3}	0.72/-0.82

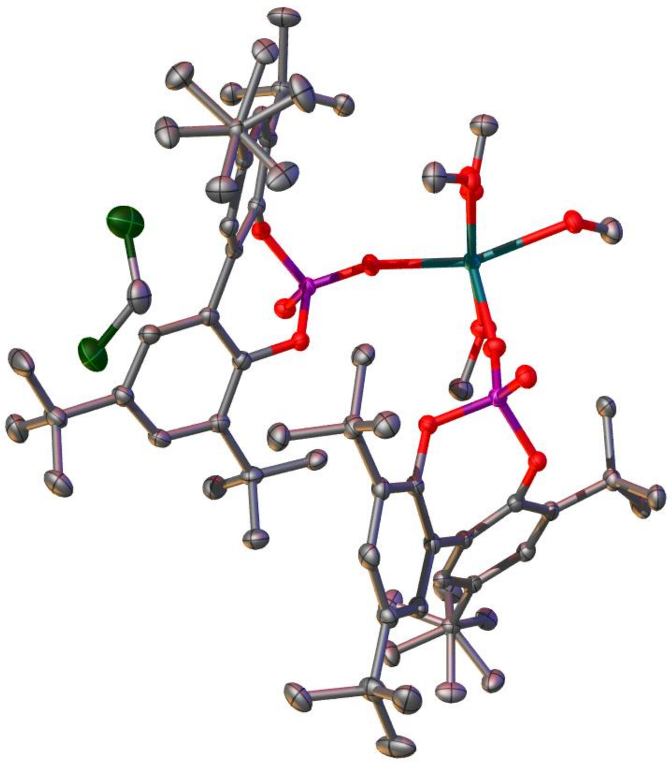

Figure S16. ORTEP drawing of complex **4**, (50% thermal ellipsoids. All hydrogen atoms are omitted for clarity).

Compound **2’** (product) – CCDC 1824281

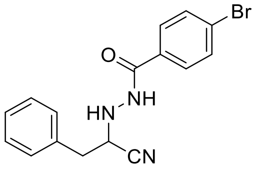

2'

Hydrogen atoms omitted for clarity in the structure shown.

The crystal structure was measured on a SuperNova, Dual, Cu at zero, AtlasS2 diffractometer. Using Olex2 [11], the structure was solved by direct methods (ShelXT) [12] and refined with ShelXL[13] using least squares minimization. The hydrogen atoms have been placed on calculated positions and were refined isotropically in a riding model. In total, seven reflexes were omitted, due to error minimization.

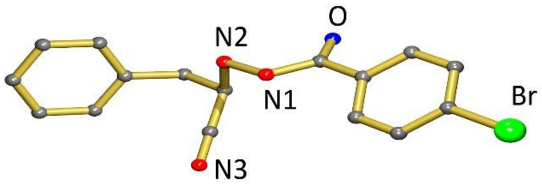

Figure S17. Crystal structure of **2’**, phenyl derivate; hydrogen atoms omitted for clarity.

Table S2. Crystal data and structure refinement for **2'**.

Identification code	hasj160209b
Empirical formula	$C_{16}H_{14}BrN_3O$
Formula weight	344.21
Temperature/K	100
Crystal system	monoclinic
Space group	C2/c
a/Å	30.5362(3)
b/Å	4.72686(4)
c/Å	19.9817(2)
α/°	90
β/°	93.6704(9)
γ/°	90
Volume/Å^3	2878.25(4)
Z	8
ρ_{calc}g/cm^3	1.589
μ/mm^{-1}	3.911
F(000)	1392.0
Crystal size/mm^3	0.5707 × 0.2216 × 0.0876
Radiation	CuKα (λ = 1.54184)
2Θ range for data collection/°	8.87 to 136.224
Index ranges	-36 ≤ h ≤ 36, -5 ≤ k ≤ 5, -22 ≤ l ≤ 24
Reflections collected	19727
Independent reflections	2636 [R_{int} = 0.0378, R_{sigma} = 0.0163]
Data/restraints/parameters	2636/150/190
Goodness-of-fit on F^2	1.101
Final R indexes [I>=2σ (I)]	R_1 = 0.0259, wR_2 = 0.0673
Final R indexes [all data]	R_1 = 0.0261, wR_2 = 0.0674
Largest diff. peak/hole / e Å^{-3}	0.36/-0.43

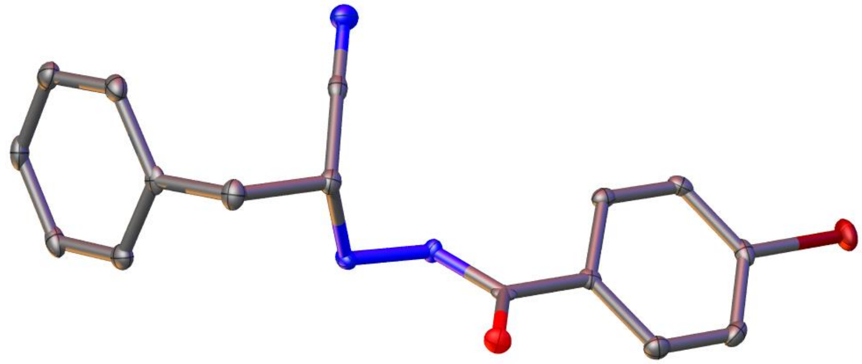

Figure S18. ORTEP drawing of compound **2’**, phenyl derivate, (50% thermal ellipsoids. All hydrogen atoms are omitted for clarity).

Tetra-*tert*-butylbiphenyl phosphoric acid **3** – CCDC 1824280

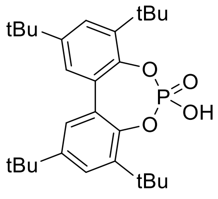

3

Hydrogen atoms omitted for clarity in the structure shown.

The crystal structure was measured on a SuperNova, Dual, Cu at zero, AtlasS2 diffractometer. Using Olex2 [11], the structure was solved by direct methods (ShelXT) [12] and refined with ShelXL [13] using least squares minimization. The hydrogen atoms have been placed on calculated positions and were refined isotropically in a riding model. Disorder of one *t*-Bu-group was refined as two positions with an approximate ration of 80:20. The co-crystalized chloroform molecule has also two disordered positions each with an approximate ration of 60:40.

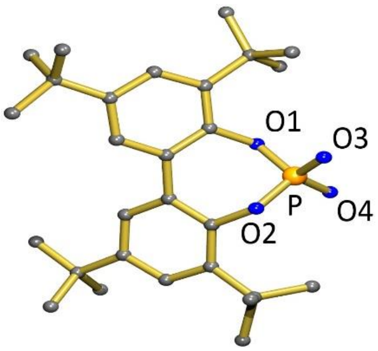

Figure S18. Crystal structure of **3**; Hydrogen atom and co-crystallized $CHCl_3$ omitted for clarity.

Table S3. Crystal data and structure refinement for **3**.

Identification code	hasj160613c
Empirical formula	$C_{29}H_{42}Cl_3O_4P$
Formula weight	591.94
Temperature/K	100.0(3)
Crystal system	monoclinic
Space group	$P2_1/n$
a/Å	15.2166(4)
b/Å	12.7596(2)
c/Å	17.8907(4)
α/°	90
β/°	112.650(3)
γ/°	90
Volume/Å^3	3205.70(14)
Z	4
ρ_{calc}g/cm^3	1.226
μ/mm^{-1}	3.300
F(000)	1256.0
Crystal size/mm^3	0.267 × 0.183 × 0.112
Crystal color	colorless
Radiation	CuKα (λ = 1.54184)
2Θ range for data collection/°	6.506 to 147.178
Index ranges	-15 ≤ h ≤ 18, -15 ≤ k ≤ 10, -18 ≤ l ≤ 21
Reflections collected	11231
Independent reflections	6193 [R_{int} = 0.0351, R_{sigma} = 0.0490]
Data/restraints/parameters	6193/0/391
Goodness-of-fit on F^2	1.039
Final R indexes [I>=2σ (I)]	R_1 = 0.0461, wR_2 = 0.1185
Final R indexes [all data]	R_1 = 0.0521, wR_2 = 0.1248
Largest diff. peak/hole / e Å^{-3}	0.70/-0.63

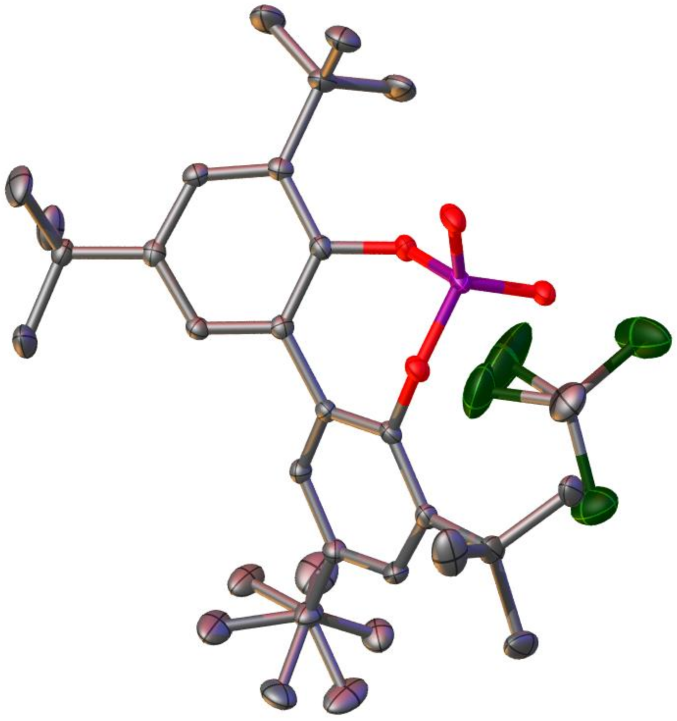

Figure S19. ORTEP drawing of compound **3** including co-crystallized $CHCl_3$ (disordered), (50% thermal ellipsoids. All hydrogen atoms are omitted for clarity.).

8. References

1 Fulmer, G. R., Miller, A. J., Sherden, N. H., Gottlieb, H. E., Nudelman, A., Stoltz, B. M., Bercaw, J. E., Goldberg, K. I., *Organometallics* **2010**, *29*, 2176-2179.

2 *Gaussian 09 Revision D.01*, Frisch, M. J., Trucks, G. W., Schlegel, H. B., Scuseria, G. E., Robb, M. A., Cheeseman, J. R., Scalmani, G., Barone, V., Petersson, G. A., Nakatsuji, H., Li, X., Caricato, M., Marenich, A. V., Bloino, J., Janesko, B. G., Gomperts, R., Mennucci, B., Hratchian, H. P., Ortiz, J. V., Izmaylov, A. F., Sonnenberg, J. L., Williams, Ding, F., Lipparini, F., Egidi, F., Goings, J., Peng, B., Petrone, A., Henderson, T., Ranasinghe, D., Zakrzewski, V. G., Gao, J., Rega, N., Zheng, G., Liang, W., Hada, M., Ehara, M., Toyota, K., Fukuda, R., Hasegawa, J., Ishida, M., Nakajima, T., Honda, Y., Kitao, O., Nakai, H., Vreven, T., Throssell, K., Montgomery Jr., J. A., Peralta, J. E., Ogliaro, F., Bearpark, M. J., Heyd, J. J., Brothers, E. N., Kudin, K. N., Staroverov, V. N., Keith, T. A., Kobayashi, R., Normand, J., Raghavachari, K., Rendell, A. P., Burant, J. C., Iyengar, S. S., Tomasi, J., Cossi, M., Millam, J. M., Klene, M., Adamo, C., Cammi, R., Ochterski, J. W., Martin, R. L., Morokuma, K., Farkas, O., Foresman, J. B., Fox, D. J., Gaussian, Inc., Wallingford CT, **2016**

3 Becke, A. D., *J. Chem. Phys.* **1993**, *98*, 5648-5652.

4 Lee, C., Yang, W., Parr, R. G., *Phys. Rev. B: Condens. Matter* **1988**, *37*, 785-789.

5 Krishnan, R., Binkley, J. S., Seeger, R., Pople, J. A., *J. Chem. Phys.* **1980**, *72*, 650-654.

6 Grimme, S., Ehrlich, S., Goerigk, L., *J. Comput. Chem.* **2011**, *32*, 1456-1465.

7 Zamfir, A., Tsogoeva, S. B., *Org. Lett.* **2010**, *12*, 188-191.

8 Liu, H., Cun, L.-F., Mi, A.-Q., Jiang, Y.-Z., Gong, L.-Z., *Org. Lett.* **2006**, *8*, 6023-6026.

9 Straub, B. F., Wrede, M., Schmid, K., Rominger, F., *Eur. J. Inorg. Chem.* **2010**, *2010*, 1907-1911.

10 Yao, L.-H., Shao, S.-X., Jiang, L., Tang, N., Wu, J.-C., *Chem. Pap.* **2014**, *68*, 1381-1389.

11 Dolomanov, O., Bourhis, L., Gildea, R., Howard, J., Puschmann, H., *J. Appl. Crystallogr.* **2009**, *42*, 339-341.

12 Sheldrick, G. M., *Acta Crystallogr. A* **2015**, *71*, 3-8.

13 Sheldrick, G. M., *Acta Crystallogr. A* **2008**, *64*, 112-122.
